# Oxaprozin-Based Amide Derivatives as Multifunctional Agents: Synthesis, Characterization, and Comprehensive Biological Profiling

**DOI:** 10.3390/pharmaceutics18101271

**Published:** 2026-10-07

**Authors:** Anđela Gogić, Miloš Nikolić, Nikola Nedeljković, Marina Vesović, Ana Živanović, Vladimir Dobričić, Jelena Bošković, Isidora Kostić, Jovana Z. Marinković, Milena Jurišević, Nevena Gajović, Bojana Simović Marković, Nebojša Zdravković, Ivan Jovanović

**Affiliations:** 1 Department of Medical Statistics and Informatics, Faculty of Medical Sciences, University of Kragujevac, Svetozara Markovića 69, 34000 Kragujevac, Serbia; andjela.gogic@fmn.kg.ac.rs (A.G.); nzdravkovic@fmn.kg.ac.rs (N.Z.); 2 Department of Pharmacy, Faculty of Medical Sciences, University of Kragujevac, Svetozara Markovića 69, 34000 Kragujevac, Serbia; milos.nikolic@fmn.kg.ac.rs (M.N.); nikola.nedeljkovic@fmn.kg.ac.rs (N.N.); ana.zivanovic@fmn.kg.ac.rs (A.Ž.); 3 Center for Molecular Medicine and Stem Cell Research, Faculty of Medical Sciences, University of Kragujevac, Svetozara Markovića 69, 34000 Kragujevac, Serbia; isidorastanisavljevic97@gmail.com (I.K.); jovanamarinkovic266@gmail.com (J.Z.M.); gajovicnevena@yahoo.com (N.G.); bojana.simovic@gmail.com (B.S.M.); ivanjovanovic77@gmail.com (I.J.); 4 Department of Pharmaceutical Chemistry, Faculty of Pharmacy, University of Belgrade, Vojvode Stepe 450, 11221 Belgrade, Serbia; vladimir.dobricic@pharmacy.bg.ac.rs (V.D.); jelena.boskovic@pharmacy.bg.ac.rs (J.B.); 5 Faculty of Medicine, University of East Sarajevo, Studentska 5, 73300 Foca, Bosnia and Herzegovina; 6 Institute of Public Health Kragujevac, Nikole Pašića 1, 34000 Kragujevac, Serbia

**Keywords:** oxaprozin, amide derivatives, cytotoxicity, immunomodulatory effect, COX-1, COX-2, molecular docking

## Abstract

**Background/Objectives:** Inflammation plays a role at multiple levels, contributing to tumorigenesis, cellular proliferation, cell survival, and metastasis. The established link between chronic inflammation and tumor development has shifted the therapeutic focus toward nonsteroidal anti-inflammatory drugs, which exert their effects primarily through cyclooxygenase inhibition. **Methods:** In the present study, the antitumor potential of novel synthesized oxaprozin derivatives was evaluated using the MTT assay. The immunomodulatory effects of the selected derivative were determined in vitro by measuring cytokine concentrations and performing flow cytometry. To investigate the inhibitory capacity of these derivatives against cyclooxygenase enzymes, an in vitro inhibition assay was conducted using fluorometric screening kits, followed by in silico validation. **Results**: All tested tumor cell lines exhibited sensitivity to compound **1**, which demonstrated the lowest cytotoxic effect against non-cancerous mMSCs and MRC-5 cells. This compound can effectively target tumor cells, inducing potent apoptosis in 4T1 breast cancer cells through a strategic shift in the Bax/Bcl-2 balance and the downregulation of the oncogenic driver Bcl-6. Although the synthesized oxaprozin derivatives showed moderate affinity for COX-1, none of the newly synthesized compounds achieved more than 50% inhibition against COX-2 at a concentration of 100 μM. Consistently, in silico procedures validated the results of the in vitro COX inhibition assay. **Conclusions:** The findings of this study indicate that compound **1** has the ability to modulate immune responses under stimulated conditions, and its potent antitumor activity may not be directly related to cyclooxygenase inhibition, suggesting the involvement of COX-independent pathways.

## 1. Introduction

Chronic inflammation is a key contributor to the development of various diseases, including cardiovascular diseases, diabetes, cancer, Alzheimer’s disease, as well as many autoimmune and rheumatic disorders [1]. In the context of cancer, inflammation plays a role at multiple levels, contributing to tumorigenesis, cellular transformation and proliferation, cell survival, and metastasis [2]. It is estimated that up to 90% of all tumors result from somatic mutations and environmental factors. Studies have demonstrated a strong link between inflammation and cancer, with two well-established mechanisms: the intrinsic pathway, in which genetic mutations that drive neoplasia also trigger inflammation within the tumor microenvironment, and the extrinsic pathway, where chronic inflammation precedes tumor development and promotes carcinogenesis [3,4,5].

The tumor microenvironment comprises both innate and adaptive immune cells, including neutrophils, macrophages, dendritic cells, myeloid-derived suppressor cells, natural killer (NK) cells, as well as T and B lymphocytes [6]. These immune cells release pro-inflammatory cytokines, such as interleukin-6 (IL-6), tumor necrosis factor-alpha (TNF-α), and interleukin-1 beta (IL-1β), which, in turn, activate key signaling pathways involving transcription factors NF-κB, AP-1, and STAT3 [2,7]. These transcription factors induce the expression of genes that promote cell proliferation, angiogenesis, and the production of additional cytokines and chemokines. Moreover, activated immune cells can either directly promote the generation of reactive oxygen species (ROS) and reactive nitrogen intermediates (RNI), leading to the formation of free radicals capable of inducing DNA damage and genomic instability, or where this effect may be mediated through the release of TNF-α [8].

One of the key enzymes involved in the inflammatory response is cyclooxygenase (COX), which exists in three isoforms: COX-1, COX-2, and COX-3. COX plays a crucial role in the metabolism of arachidonic acid. Since its discovery, COX-2 has been recognized as an important mediator of pain and is a primary target of anti-inflammatory therapy [9]. Its main function is the synthesis of prostaglandin E2 (PGE2), a bioactive lipid involved in regulating various carcinogenic pathways [10]. Unlike COX-1, which is constitutively expressed in many tissues, COX-2 is an inducible enzyme with generally low expression under normal physiological conditions, except in certain cells such as those in the kidneys, gastrointestinal tract, central nervous system, and female reproductive system. However, COX-2 is frequently overexpressed in tumorigenic regions of various cancer types, including adenocarcinoma, hepatocellular carcinoma, squamous cell carcinoma (SCC), cholangiocarcinoma, transitional cell carcinoma, and endometrial carcinoma, where it is involved in proliferation, angiogenesis, and resistance to apoptosis primarily through the PGE2/EP4 signaling pathway [11]. It also induces epithelial–mesenchymal transition (EMT) which is a promotor of cancer invasiveness [10].

The established link between chronic inflammation and tumor development, along with the role of cyclooxygenase as a key enzyme in both the inflammatory response and tumorigenesis, has shifted the therapeutic focus toward nonsteroidal anti-inflammatory drugs (NSAIDs), which exert their effects primarily through cyclooxygenase inhibition [12,13].

Oxaprozin (4,5-diphenyl-2-oxazolepropionic acid) is an achiral non-steroidal anti-inflammatory drug and a propionic acid derivative widely used for the treatment of inflammatory diseases. Oxaprozin plays an important role in the control of pain and inflammation by inhibiting both cyclooxygenase enzymes and prostaglandin synthesis [14,15]. The efficacy of oxaprozin in the treatment of inflammatory diseases, particularly rheumatoid arthritis, is also a result of significant PPARγ-related and NF-κB-dependent synoviocyte apoptosis [16]. Another COX-independent mechanism of action of oxaprozin is based on the inhibition of anandamide catabolism by competing with its natural substrate and thereby reducing the transmission of nociceptive signals to the brain [17]. Oxaprozin has been shown to be effective at therapeutic doses in relieving symptoms and reducing infections in patients with contact dermatitis. It inhibits the enzymes spleen tyrosine kinase, zeta-associated protein 70, and phosphodiesterase IV [18]. Recent studies also suggest the possibility that oxaprozin modulates the activity of matrix metalloproteinase 9, which is important in the suppression of innate antitumor immunity [19].

Although these are of great importance due to their analgesic, antipyretic, and anti-inflammatory effects, the therapeutic use of NSAIDs is limited by their gastrointestinal (GI) side effects. One possible solution to overcome these side effects is the “prodrug” approach. This strategy is based on the design of inactive compounds that are converted into an active compound by metabolic reactions, with the aim of improving the pharmacological activity of the parent drug [20,21]. Chronic oral use of NSAIDs can lead to perforation of the GI tract due to local irritation caused by the carboxyl group of the drug. Therefore, masking the carboxyl group by converting it to esters, thioesters, amides, and carbamates is a successful prodrug approach in overcoming GI complications [22].

Peesa and colleagues synthesized the prodrug compound by esterifying oxaprozin with a biodegradable carrier, dextran. The resulting derivative showed similar therapeutic efficacy to oxaprozin, with a lower incidence of GI side effects in experimental animals [22]. The introduction of antioxidant groups may enhance the anti-inflammatory effect of the drug. Therefore, oxaprozin-paeonol ester was synthesized by utilizing the anti-inflammatory and antioxidant properties of paeonol to mask the carboxylic acid group of oxaprozin. This ester showed remarkable anti-inflammatory activity in an ear edema model in mice, including histologic changes and release of pro-inflammatory cytokines, which are related to its ability to decrease NF-κB activation by blocking Akt/IKK activity [23]. A study conducted by Valuri and colleagues showed that oxaprozin acylhydrazone derivatives containing aldehyde substituents such as *p*-nitro, 2,5-dimethoxy, and 3,4,5-trimethoxy exhibited significant analgesic and anti-inflammatory effects compared to the reference compound [24]. Furthermore, based on the collected data, oxaprozin-1,3,4-oxadiazole derivatives have shown notable anticancer and antibacterial activity against A549 and *Klebsiella pneumoniae*, along with a good safety profile against the normal human HEK293T cell line [25]. A study on new 3,5-disubstituted 1a,3,4-oxadiazole derivatives showed that the introduction of unsaturation, particularly allyl and propargyl groups, reduced activity against the prostate adenocarcinoma cell lines PPC-1 and the lung adenocarcinoma cell lines HTB-57, while the compound with a 4-methoxybenzyl substituent showed promising activity [26].

COX-2 might contribute to carcinogenesis by inhibiting apoptosis and promoting angiogenesis [27]. Based on these findings, various drug candidates have been synthetized to target COX-2. In addition to COX-2 inhibition, it has been shown that some of these compounds also target lipoxygenase (LOX), epidermal growth factor receptor (EGFR), vascular endothelial growth factor receptor (VEGFR-2), topoisomerases, and other molecules [27]. For example, licofelone inhibits COX and LOX and consequently induces apoptosis in HCA-7 cells [28]. Quinazolinone-based derivatives inhibit EGFR, VEGFR-2, and COX-2 and induce apoptosis and G2/M cell cycle arrest in MCF-7 cells [29]. Furthermore, pyrazole/1,2,4-triazole derivates inhibit COX and EGFR and induce apoptosis in MCF-7 cells by increasing Bax expression and decreasing Bcl-2 expression [30]. Pyrazole-based COX-2, EGFR, and Topo-1 inhibitors reduced the viability of MCF-7 and HT-29 cells, while inducing apoptosis by changing the Bax/Bcl-2 ratio [31]. New nimesulide-derived urea reduced the viability of 4T1, MDA-MB-231, and MCF-7 cells, increased the percentage of late apoptotic cells, and inhibited MetAP type II [32]. Newly synthetized analogs of celecoxib/dasatinib inhibited COX-2, HER-2, and EGFR and reduced the viability of MCF-7 cells [33]. However, some findings indicate that COX-2 inhibition is not always the mechanism responsible for the antitumor effects of NSAID-based compounds. Two COX-2 inhibitor derivatives induced apoptosis in MCF-7 cells, increased the Bax/Bcl-2 ratio, and activated caspase-3 through inhibition of NF-kB, FHC, and ERK signaling [34]. These findings indicated that their antiproliferative effects were not associated with COX-2 expression. Therefore, modification of NSAIDs and their incorporation into hybrid molecules may lead to compounds with additional anticancer properties. Hybrid compounds may act against cancer cells through different mechanisms. For example, carbazole-thiazolidinone hybrids significantly reduced the viability of leukemia cells, decreased c-MYC expression, and possibly affected β-catenin signaling [35].

Inspired by the above findings, the present study aimed to synthesize novel oxaprozin derivatives designed to modulate interactions with the COX enzymes through structural modification of the carboxyl group. The newly synthesized compounds were evaluated for their anticancer potential by assessing their effects using the MTT assay and an immunomodulatory model. Furthermore, the selective binding profiles of five novel oxaprozin derivatives toward COX-1 and COX-2 were investigated using complementary in vitro and in silico approaches.

## 2. Materials and Methods

### 2.1. Chemicals and Instruments

All chemicals and solvents were of commercial grade and used without further purification. Oxaprozin, methyl 4-aminobutyrate hydrochloride, and triethylamine (TEA) were obtained from Acros Organics (Geel, Belgium). L-alanine methyl ester hydrochloride, tetrahydrofuran (THF), 1-hydroxybenzotriazole (HOBt), and *N*,*N*-dimethylformamide (DMF) were purchased from Sigma-Aldrich (Steinheim, Germany). L-tryptophan methyl ester hydrochloride and beta-alanine methyl ester hydrochloride were obtained from TCI (Tokyo, Japan). 1-Ethyl-3-(3-dimethylaminopropyl)carbodiimide hydrohloride (EDC-HCl) was purchased from Carl Roth (Karlsruhe, Germany); chloroform and methanol were bought from JT Baker (Loughborough, UK); while silica gel for preparative thin-layer chromatography (TLC) was obtained from Merck (Darmstadt, Germany).

The synthesized oxaprozin derivatives were structurally characterized by determining melting points and by spectroscopic methods (IR, NMR, HRMS, and MS/MS). Melting points were determined using the Boetius PHMK 05 apparatus (Radebeul, Germany). IR spectra were recorded using the Nicolet iS10 FT-IR spectrometer (Thermo Fisher Scientific, Madison, WI, USA). The ^1^H NMR and ^13^C NMR spectra were recorded on a Bruker Avance III NMR spectrometer (Bruker Biospin GmbH, Rheinstetten, Germany), operated at 400 MHz (^1^H NMR) and 100 MHz (^13^C NMR). Exact masses were determined using the LTQ Orbitrap XL mass spectrometer (Thermo Fisher Scientific, Bremen, Germany). MS/MS analyses were performed using the TSQ Quantum Access MAX triple quadrupole mass spectrometer (Thermo Fisher Scientific, San Jose, CA, USA), equipped with a heated electrospray ionization source (HESI).

### 2.2. Synthetic Procedures

Oxaprozin derivatives were synthesized according to an adapted procedure from the literature [36]. Oxaprozin (59 mg, 0.2 mmol, 1 eq) was dissolved in DMF (4 mL), and this solution was stirred at room temperature for 30 min. Subsequently, EDC (69 mg, 0.36 mmol, 1.8 eq) and HOBt (40.5 mg, 0.3 mmol, 1.5 eq) were introduced into the reaction mixture. The corresponding methyl ester of amino acid hydrochloride (0.2 mmol; methyl ester of L-alanine, methyl ester of β-alanine, methyl ester of 4-aminobutanoic acid, methyl ester of L-tryptophan, methyl ester of 8-aminooctanoic acid) was dissolved in DMF (2 mL), followed by the addition of triethylamine (TEA, 54 μL, 0.4 mmol, 2 eq). The mixture of oxaprozin, ECC, and HOBt was added dropwise to a previously prepared amino acid ester solution. The reaction was maintained on a magnetic stirrer at room temperature for 5–6 h, after which the mixture was left overnight at a temperature not exceeding 8 °C. Thereafter, the resulting solution was evaporated to dryness in the digester at room temperature overnight. After the evaporation, liquid–liquid extraction was carried out in three steps using chloroform and three parts of polar solvent (water). After re-evaporation to dryness, the residues were dissolved in the mixture of chloroform and methanol and purified using preparative thin-layer chromatography. The mobile phases used for preparative TLC were chloroform/methanol 95:5 (*v*/*v*) (compounds **1**, **2**, and **3**) and chloroform/methanol 97:3 (*v*/*v*) (compounds **4** and **5**).

*Methyl (3-(4,5-diphenyloxazol-2-yl)propanoyl)alaninate*, C_22_H_22_N_2_O_4_ (**1**)

Yield: 39.64%. White crystalline solid. Melting point: 116.1–118.5 °C. IR (ATR) ν_max_ (cm^−1^): 690.33, 758.25, 1213.93, 1556.24, and 1747.97. **^1^H NMR (400 MHz, CDCl_3_) δ ppm** 1.398 (3H, d, *J* = 7.2 Hz, CHC**H_3_**), 2.814 (2H, t, *J* = 7.4 Hz OXCH_2_C**H_2_**), 3.211 (2H, t, *J* = 7.4 Hz OXC**H_2_**CH_2_), 3.72 (3H, s, OC**H_3_**), 4.62 (1H, q, *J* = 7.3 Hz, C**H**CH_3_), 6.564 (1H, d, *J* = 7.2 Hz, CON**H**), 7.315–7.623 (10H, m, ArH). **^13^C NMR (100 MHz, CDCl_3_) δ ppm** 18.49 (CH**C**H_3_), 23.86 (OX**C**H_2_CH_2_), 32.91 (OXCH_2_**C**H_2_), 48.16 (**C**HCH_3_), 52.44 (O**C**H_3_), 126.50, 127.88, 128.08, 128.49, 128.56, 128.64, 128.91, 132.43, 135.00, 145.48, 162.22 (aromatic carbons), 170.77 (**C**ONH), 173.41 (**C**OOR). *m*/*z* = 379.0, [M+H]^+^, 346.89, 275.90, 233.93, 205.96, 117.14, and 103.15. MS [M+H]^+^ calculated = 379.16523; observed = 379.16454.

*Methyl 3-(3-(4,5-diphenyloxazol-2-yl)propanamido)propanoate*, C_22_H_22_N_2_O_4_ (**2**)

Yield: 26.42%. White powder. Melting point: 78.3–79.9 °C. IR (ATR) ν_max_ (cm^−1^): 696.35, 766.57, 1224.43, 1636.09, and 1730.46. **^1^H NMR (400 MHz, CDCl_3_) δ ppm** 2.540 (2H, t, *J* = 5.8 Hz, CONHCH_2_C**H_2_**), 2.754 (2H, t, *J* = 7.4 Hz, OXCH_2_C**H_2_**), 3.197 (2H, t, *J* = 7.4 Hz, OXC**H_2_**CH_2_), 3.553 (2H, t, *J* = 6.0 Hz, CONHC**H_2_**CH_2_), 3.643 (3H, s, OC**H_3_**), 6.444 (1H, t, CON**H**), 7.314–7.616 (10H, m, ArH). **^13^C NMR (100 MHz, CDCl_3_) δ ppm** 23.94 (OX**C**H_2_CH_2_), 33.04 (CONHCH_2_**C**H_2_), 33.85 (OXCH_2_**C**H_2_), 34.91 (CONH**C**H_2_CH_2_), 51.78 (O**C**H_3_), 126.47, 127.91, 128.07, 128.47, 128.56, 128.64, 128.93, 132.46, 135.02, 145.44, 162.27 (aromatic carbons), 171.17 (**C**ONH), 173.00 (**C**OOR). *m*/*z* = 379.0, [M+H]^+^, 378.25, 346.70, 275.92, 233.97, 205.98, and 103.15. MS [M+H]^+^ calculated = 379.16523; observed = 379.16508.

*Methyl 4-(3-(4,5-diphenyloxazol-2-yl)propanamido)butanoate*, C_23_H_24_N_2_O_4_ (**3**)

Yield: 48.41%. White powder. Melting point: 70.6–71.9 °C. IR (ATR) ν_max_ (cm^−1^): 687.46, 763.18, 1168.62, 1636.50, and 1735.34. **^1^H NMR (400 MHz, CDCl_3_) δ ppm** 1.825 (2H, quint, *J* = 6.9 Hz, CONHCH_2_C**H_2_**CH_2_), 2.325 (2H, t, *J* = 7.2 Hz, CONHCH_2_CH_2_C**H_2_**), 2.749 (2H, t, *J* = 7.2 Hz, OXCH_2_C**H_2_**), 3.198 (2H, t, *J* = 7.2 Hz, CONHC**H_2_**CH_2_CH_2_), 3.314 (2H, t, *J* = 6.4 Hz, OXC**H_2_**CH_2_), 3.631 (3H, s, OC**H_3_**), 6.304 (1H, t, CON**H**), 7.312–7.619 (10H, m, ArH). **^13^C NMR (100 MHz, CDCl_3_) δ ppm** 24.02 (OX**C**H_2_CH_2_), 25.6 (CONHCH_2_**C**H_2_CH_2_), 31.38 (CONHCH_2_CH_2_**C**H_2_), 33.94 (OXCH_2_**C**H_2_), 39.00 (CONH**C**H_2_CH_2_CH_2_), 51.64 (O**C**H_3_), 126.46, 127.87, 128.09, 128.49, 128.56, 128.63, 128.86, 132.40, 134.95, 145.48, 162.38 (aromatic carbons), 171.38 (**C**ONH), 173.74 (**C**OOR). *m*/*z* = 393.2, [M+H]^+^, 275.99, 234.03, 206.03, 117.05, 103.15, and 101.12. MS [M+Na]^+^ calculated = 415.16283; observed = 415.16204.

*Methyl (3-(4,5-diphenyloxazol-2-yl)propanoyl)tryptophanate*, C_30_H_27_N_3_O_4_ (**4**)

Yield: 45.49%. White powder. Melting point: 72.3–76.2 °C. IR (ATR) ν_max_ (cm^−1^): 693.40, 742.15, 763.06, 1212.42, and 1655.79. **^1^H NMR (400 MHz, CDCl_3_) δ ppm** 2.77 (2H, t, *J* = 7.2 Hz, OXCH_2_C**H_2_**), 3.180 (2H, d, *J* = 7.6 Hz, CONHCHC**H_2_**), 3.312 (2H, t, *J* = 8.0 Hz, OXC**H_2_**CH_2_), 3.655 (3H, s, OC**H_3_**), 4.964 (1H, t, *J* = 6.2 Hz, CONHCHC**H_2_**), 6.907 (1H, d, CON**H**), 7.077–7.589 (15H, m, ArH), 7.604 (1H, d, *J* = 2.4 Hz, indole-N**H**). **^13^C NMR (100 MHz, CDCl_3_) δ ppm** 23.71 (OX**C**H_2_CH_2_), 27.50 (CONHCH**C**H_2_), 32.77 (OXCH_2_**C**H_2_), 52.35 (O**C**H_3_), 52.97 (CONH**C**HCH_2_), 109.85, 111.16, 118.47, 119.62, 122.15, 122.93, 126.46, 127.56, 128.05, 128.48, 128.58, 128.66, 128.89, 132.59, 134.91, 135.94, 145.50, 162.27 (aromatic carbons), 170.84 (**C**OOR), 172.25 (**C**ONH). *m*/*z* = 494.1, [M+H]^+^, 462.07, 275.94, 233.94, 205.96, 160.00, and 103.07. MS [M+Na]^+^ calculated = 516.18938; observed = 516.19030.

*Methyl 8-(3-(4,5-diphenyloxazol-2-yl)propanamido)octanoate*, C_27_H_32_N_2_O_4_ (**5**)

Yield: 55.73%. White crystalline solid. Melting point: 85.7–86.5 °C. IR (ATR) ν_max_ (cm^−1^): 687.20, 757.02, 1167.82, 1522.90, and 1671.66. **^1^H NMR (400 MHz, CDCl_3_) δ ppm** 1.273 (6H, s, CONHCH_2_CH_2_C**H_2_**C**H_2_**C**H_2_**CH_2_CH_2_), 1.489–1.510 (2H, m, CONHCH_2_CH_2_CH_2_CH_2_CH_2_C**H_2_**CH_2_), 1.567–1.602 (2H, m, CONHCH_2_C**H_2_**CH_2_CH_2_CH_2_CH_2_CH_2_), 2.276 (2H, t, *J* = 7.4 CONHCH_2_CH_2_CH_2_CH_2_CH_2_CH_2_C**H_2_**), 2.578 (2H, t, *J* = 7.1 OXCH_2_C**H_2_**), 3.126–3.145 (2H, m, CONHC**H_2_**CH_2_CH_2_CH_2_CH_2_CH_2_CH_2_), 3.230–3.287 (2H, m, OXC**H_2_**CH_2_), 3.665 (3H, s, OC**H_3_**), 5.571 (1H, t, CON**H**), 7.374–7.864 (10H, m, ArH). **^13^C NMR (100 MHz, CDCl_3_) δ ppm** 23.86 (OX**C**H_2_CH_2_), 24.80 (CONHCH_2_CH_2_CH_2_CH_2_CH_2_**C**H_2_CH_2_), 26.66 (CONHCH_2_CH_2_**C**H_2_CH_2_CH_2_CH_2_CH_2_), 28.90 (CONHCH_2_CH_2_CH_2_CH_2_**C**H_2_CH_2_CH_2_), 29.18 (CONHCH_2_**C**H_2_CH_2_CH_2_CH_2_CH_2_CH_2_), 29.35 (CONHCH_2_CH_2_CH_2_**C**H_2_CH_2_CH_2_CH_2_), 33.97 (CONHCH_2_CH_2_CH_2_CH_2_CH_2_CH_2_**C**H_2_), 33.99 (OXCH_2_**C**H_2_), 39.73 (CONH**C**H_2_CH_2_CH_2_CH_2_CH_2_CH_2_CH_2_), 51.45 (O**C**H_3_), 126.78, 126.93, 128.00, 128.51, 128.65, 128.74, 129.13, 129.20, 129.33, 130.57, 130.76 (aromatic carbons), 167.91 (**C**ONH), 174.22 (**C**OOR). *m*/*z* = 449.1, [M+H]^+^, 416.94, 275.82, 274.73, 233.90, 205.94, and 103.13. MS [M+H]^+^ calculated = 449.24348; observed = 449.24277.

### 2.3. MTT Assay

MTT assay was performed in order to examine the cytotoxicity of the tested compounds according to previously published protocol [37]. Cytotoxic effects were determined toward a panel of cancer cell lines: mouse breast carcinoma (4T1, CRL-2539), mouse colorectal carcinoma (CT26, CRL-2638), human colorectal carcinoma (HCT116, CCL-247), and human breast carcinoma (MCF-7, HTB-22). Also, mouse mesenchymal stem cells (mMSC) and human fibroblasts (MRC-5, CCL-171) were used as noncancer cell lines. All cell lines were obtained from ATCC, except mMSCs (S1502-100), which were purchased from Gibco. Cells were grown in complete Dulbecco’s Modified Eagle Medium (DMEM) under standard conditions (5% CO_2_, 37 °C). Cells were seeded into 96-well plates (density of 5 × 10^3^ cells/well). Cells were allowed to grow and attach, following treatment with tested compounds in a concentration range of 125–0.98 μM for 48 h. The tested compounds were dissolved in DMSO, diluted with complete DMEM, and the final concentration of DMSO was less than 0.05% (*v*/*v*) per well in all experiments. Following incubation, MTT solution was added, and after the incubation period, optical density was determined using the Zenyth 3100 microplate multimode detector (Salzburg, Austria). Cell viability was expressed as a percentage of the control according to the formula from the literature [38], and IC_50_ values were calculated. All experiments were performed in triplicate.

### 2.4. Flow Cytometry Analysis

After treatment with 94 μM of compound **1**, or after growing in DMEM (supplemented with 0.05% of DMSO-control 4T1 cells) for 24 h, 4T1 cells were stained with Annexin-V-FITC and propidium iodide (PI) (BD Pharmingen, San Diego, CA, USA), as previously described, and flow cytometry was performed [38]. Also, the same experimental groups in additional experiments were used in order to perform flow cytometry analysis after incubation with antibodies specific for Bax (Abcam Cambridge, Cambridge, UK), Bcl-2 (Abcam Cambridge, UK), Bcl-6 (Thermo Fisher Scientific, Waltham, MA, USA), caspase-3 (Abcam Cambridge, UK), Ki-67 (eBioscience, San Diego, CA, USA), cyclin D (Abcam Cambridge, UK), p21 (Abcam Cambridge, UK), p27 (Abcam Cambridge, UK), pan-Akt (Thermo Fisher Scientific, Waltham, MA, USA), and secondary antibodies, as previously described [38,39]. Also, a FACSCalibur Flow Cytometer (BD Biosciences, San Jose, CA, USA) was used, and the data were analyzed using FlowJo (version 10.7.2; Tree Star Inc., Ashland, OR, USA).

### 2.5. Isolation and Treatment of Splenocytes

Splenocytes were isolated from spleens removed from euthanized healthy BALB/c mice. After excision, the spleens were washed in phosphate-buffered saline (PBS) and mechanically homogenized in RPMI-1640 medium to obtain a single-cell suspension. Cell suspensions were passed through a 40 μm nylon cell strainer to eliminate tissue fragments and aggregates. After centrifugation (1500 rpm, 5 min, 4 °C), erythrocytes were lysed using RBC lysis buffer for 5 min on ice. The lysis reaction was stopped by the addition of RPMI-1640 medium, followed by an additional centrifugation step under the same conditions. The resulting cell pellet was resuspended in complete RPMI-1640 medium, supplemented with 10% fetal bovine serum (FBS), 2 mmol/L L-glutamine, 1 mmol/L penicillin, and 1 mmol/L streptomycin. Cell numbers and viability were assessed using the trypan blue assay. Only cells with viability exceeding 90% were included in subsequent experiments [40].

### 2.6. Evaluation of Immunomodulatory Effect of Compound ***1***, In Vitro

Splenocytes from previously healthy BALB/c mice were seeded in 96-well plates at a density of 2 × 10^5^ cells per well and divided into experimental groups: control cells (DMEM with 0.05% of DMSO), cells stimulated with *Escherichia coli* lipopolysaccharide (LPS, 0.5 μg/mL), cells stimulated with Concanavalin A (ConA, 0.5 μg/mL), cells treated with compound **1** alone, cells co-treated with ConA and compound **1**, and cells co-treated with LPS and compound **1** [41,42]. The concentration of compound **1** was selected based on the previously determined IC_50_ value. The cultures were maintained for 24 h at 37 °C in a humidified incubator containing 5% CO_2_. After the incubation period, cell viability was tested by trypan blue assay and remained above 90% in all wells, and there were no differences between the groups.

### 2.7. Cytokine Analysis

Following incubation, culture supernatants were harvested after centrifugation (1500 rpm, 10 min, 4 °C) to remove residual cells and debris. Concentrations of tumor necrosis factor-α (TNF-α), interleukin (IL)-1β, interferon (IFN)-γ, IL-17, and IL-10 were quantified using commercially available enzyme-linked immunosorbent assay (ELISA) kits (R&D Systems, Minneapolis, MN, USA), according to the manufacturer’s recommendations [43]. Optical density was measured at 450 nm, and cytokine concentrations were determined using standard calibration curves generated for each analysis.

### 2.8. In Vitro Investigation of COX-1 and COX-2 Inhibitory Activity

To assess the ability of the tested derivatives to inhibit COX-1 and COX-2 activity, we conducted an in vitro COX inhibition study using fluorometric cyclooxygenase inhibitor screening kits (Cat. No. ab204698 and ab283401, respectively) (Abcam, Cambridge, UK). These assays measure fluorescent signals produced by prostaglandin G2, an intermediate product generated by the activity of COX enzymes, and quantify COX inhibitory activity. All experiments were performed according to the manufacturer’s instructions [44].

All tested samples were prepared at a concentration of 5 mM in DMSO and subsequently diluted with the same solvent to obtain the test solutions. The next step involved further dilution of the test compounds with assay buffer. Namely, 2 μL of each test solution was mixed with 8 μL of COX assay buffer. The results are presented as the percentage inhibition of enzyme activity, where the solvent control (SC) was obtained by mixing DMSO (2 μL) and the COX assay buffer (8 μL); the enzyme control (EC) contained only COX assay buffer (10 μL), while the samples (S) included 10 μL of the diluted test solutions. After necessary preparation, all samples were placed in separate wells of the assay plate. The percentage inhibition of enzyme activity was calculated based on the following equation:

(1) $$\%\;Inhibition=\left(\frac{Absorbance\;of\;EC-Absorbance\;of\;S}{Absorbance\;of\;EC}\right)\times 100$$

Kinetic fluorescence measurements were employed to monitor the change in fluorescence intensity of the samples over a 10-min period at 25 °C using excitation and emission wavelengths of 535 nm and 587 nm, respectively. The Synergi LX multimode microplate reader (BioTek, Shoreline, WA, USA) was used to estimate fluorescence measurements.

### 2.9. In Silico Molecular Docking Simulation

The binding potential of the synthesized compounds was evaluated by molecular docking simulation, targeting the active sites of COX enzymes. The study was performed using AutoDock Vina software 1.1.2 [45], while 3D structures of oxaprozin derivatives were optimized by MM2 force fields from Chem3D Ultra 7.0 [46].

The crystal structures of the molecular targets COX-1 (ID: 5WBE) and COX-2 (ID: 1CX2) were downloaded from the Protein Data Bank (PDB) (Table 1). BIOVIA Discovery Studio Visualizer [47] was used for the preparation of target enzymes by removing water molecules, unnecessary chains, and co-crystallized ligands. Subsequently, the protein structures underwent a process in AutoDock Tools [48] that involved the addition of polar hydrogen atoms and Kollman charges. The docking simulation was performed by fitting the flexible structures of oxaprozin derivatives into the rigid structures of target enzymes. The coordinates were determined based on the locations of co-crystallized ligands in COX-1 and COX-2, and the search area was defined as a grid box measuring 28 × 28 × 28 Å^3^ for both enzymes, with a grid spacing of 0.375 Å. Finally, visualization of the best-docked conformations of the tested molecules was presented using PyMOL 2.4.1 [49].

Validation of the docking process was assessed by docking the co-crystallized ligands to the enzyme and comparing their positions with the crystal structure. The Root Mean Square Deviation (RMSD) was calculated to estimate the validity of the docking procedure.

## 3. Results and Discussion

### 3.1. General Procedure for the Synthesis of Oxaprozin Derivatives

In continuation of our prior investigations, the aim of this study was the synthesis and biological evaluation of novel oxaprozin derivatives. The design of the synthesized compounds was based on structure–activity relationship data reported for various anti-inflammatory agents in the literature [23,24,25,26]. Selection of amino acid esters for the synthesis process was guided by their different steric and electronic characteristics, which allowed for the examination of their influence on the inhibition of COX enzymes.

**Scheme 1:**
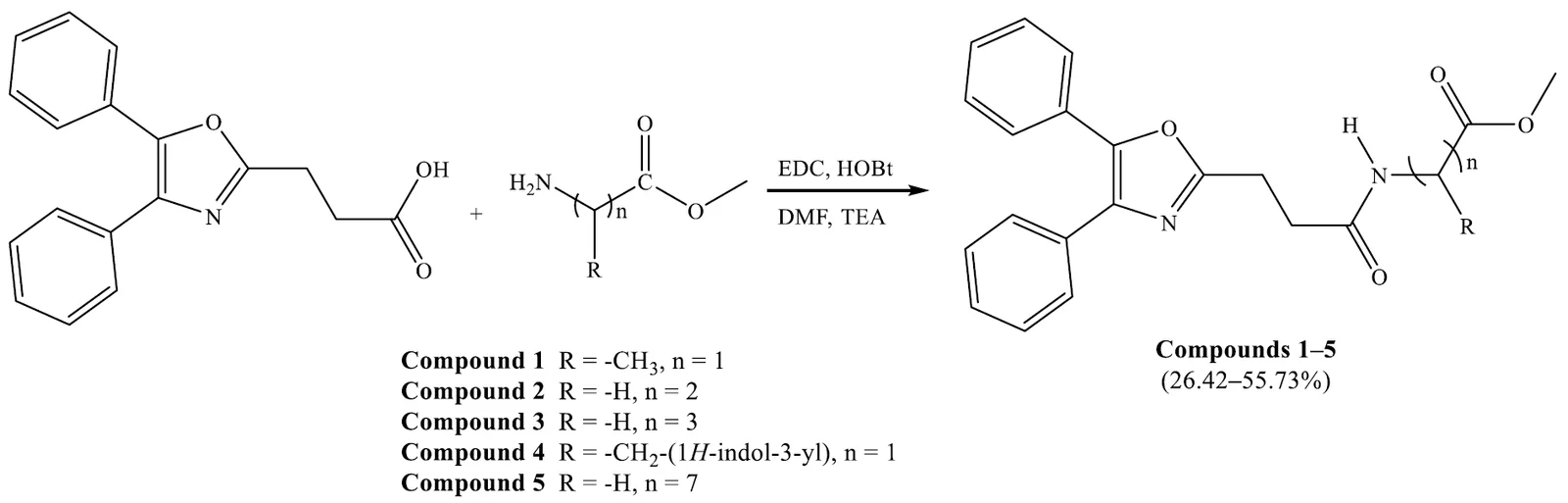
Synthesis of investigated oxaprozin derivatives.

The synthetic pathway includes conversion of the free carboxyl group into an amide moiety using coupling agents EDC and HOBt with the addition of the corresponding amino acid ester and DMF as a solvent. A series of compounds was synthesized, as shown in Scheme 1**.** The progress of the synthesis was followed by the TLC technique, while structures of compounds were determined and confirmed by melting points and spectroscopic methods (IR, NMR, HRMS, and MS/MS).

### 3.2. Cytotoxicity of Oxaprozin Derivatives

In order to determine the effects of newly synthetized derivatives of oxaprozin on the viability of cancer and noncancer cells, an MTT assay was performed. Mouse breast carcinoma (4T1), mouse colorectal carcinoma (CT26), human breast carcinoma (MCF-7), human colorectal carcinoma (HCT116), mouse mesenchymal stem cells (mMSC), and human fibroblasts (MRC-5) were treated with different concentrations of oxaprozin derivatives (0.98–125 μM) for 48 h under standard conditions. The activity of derivatives was also compared to oxaprozin. Based on the results of the MTT assay, IC_50_ values were calculated and are presented in Table 2.

All tested compounds decreased the viability of all tested cell lines in a dose-dependent manner at the higher tested concentrations (Figure S1). The mouse breast cancer cell line, 4T1, seemed to be the most sensitive in the presence of compounds **1**, **2**, and **4**, with IC_50_ values of 94.06 μM, 91.17 μM, and 88.73 μM, respectively. The highest cytotoxic capacity was observed for compounds **1**, **4**, and **5** toward mouse colon cancer CT26 cells, with slightly higher IC_50_ values of 115.21 μM, 101.13 μM, and 100.48 μM, respectively. Compounds **1**, **2**, and **4** showed the highest cytotoxic activity toward MCF-7 cells, while HCT116 cells were most sensitive to compounds **1**, **4**, and **5**. The other studies also reported cytotoxic activity of oxaprozin derivatives against the A549 cell line [25] and HCT-15 cell line [24]. Interestingly, although all tested tumor cell lines were sensitive to the action of compound **1**, this oxaprozin derivative showed the lowest cytotoxic effect toward noncancer mMSC and MRC-5 cells (Table 2). The selectivity index (SI), which indicates the specificity of the tested compound towards cancer cells, was calculated as the ratio of IC_50_ values for mouse or human noncancer cells vs. mouse or human cancer cells (Table 3). The cytotoxic potential of compound **1** was approximately two times higher in cancer cells than in noncancer cells (Table 3), indicating higher selectivity. Due to its highest selectivity, indicating a potentially better safety profile, compound **1** was selected for further experiments.

### 3.3. Compound ***1*** Induces Apoptosis in 4T1 Cells

**Figure 1:**
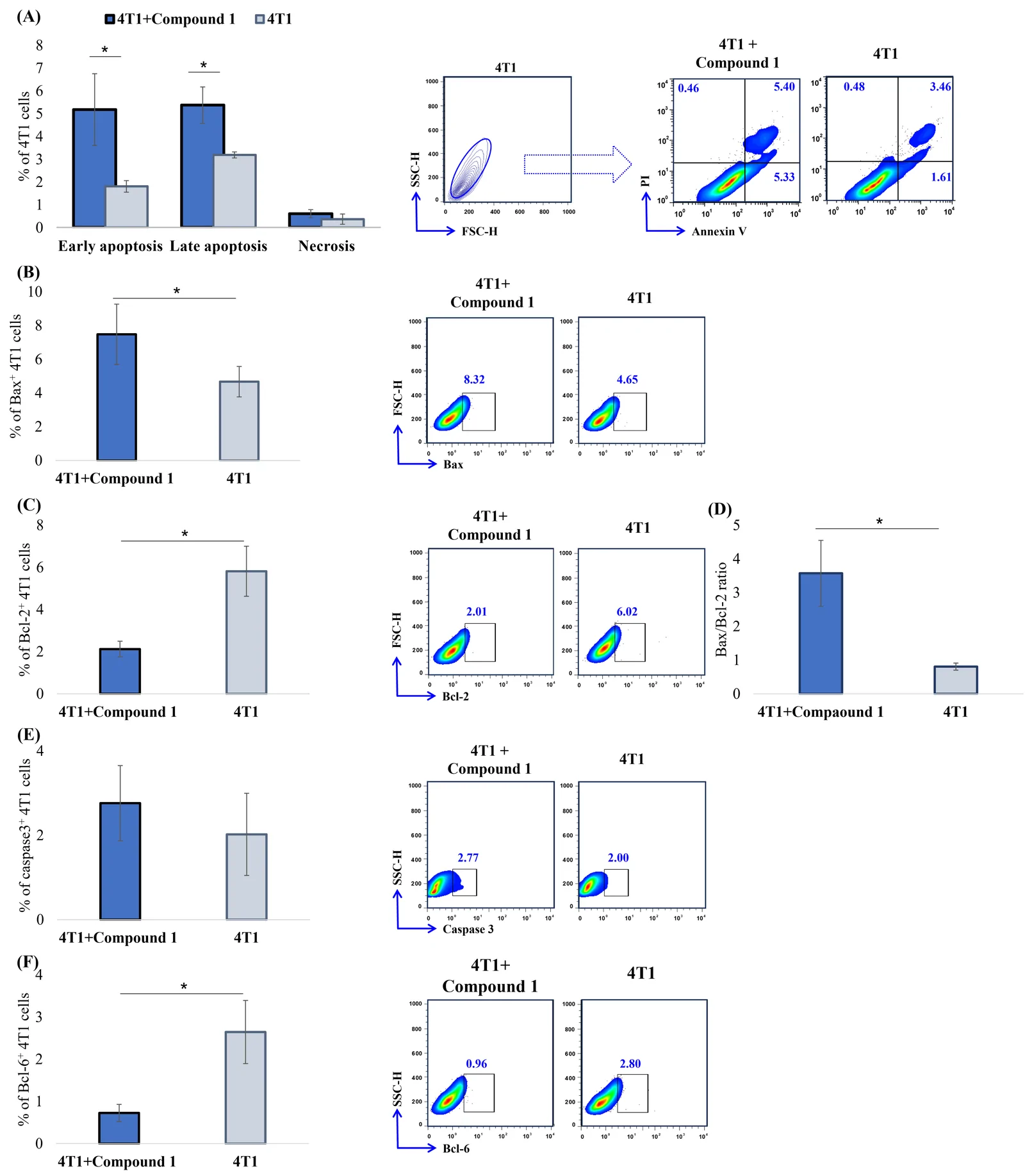
Compound **1** induced apoptosis in 4T1 cells. After Annexin-V (FITC) and PI double staining, rates of compound **1**-treated (94 μM for 24 h) as well as control 4T1 cells were determined by flow cytometry (**A**). Analysis of the percentage of Bax^+^ (**B**), Bcl-2^+^ (**C**), caspase-3^+^ (**E**), Bcl6^+^ (**F**), and 4T1 cells exposed to compound **1** (94 μM for 24 h) using flow cytometry. Also, the Bax/Bcl-2 ratio was determined (**D**). Representative dot plots are presented. The data are shown as means ± SDs of 3 independent experiments. Mann–Whitney U test: * *p* < 0.05 compared with the untreated group.

Further, we investigated the possible mechanisms implicated in compound **1**-induced inhibition of 4T1 cell viability. Murine breast cancer 4T1 cells were treated with compound **1** for 24 h and stained with Annexin-V and propidium iodide. As illustrated in Figure 1A, compound **1** treatment significantly increased the percentage of AnnV^+^/PI^−^ and AnnV^+^/PI^+^ 4T1 cells, compared to control 4T1 cells, indicating early and late apoptosis, respectively. Apoptosis is one of the key events in cancer progression, and many chemotherapeutic compounds induce apoptotic cell death as their primary mechanism of action. It is well known that apoptosis could be regulated by the family of Bcl-2 proteins [50]. These proteins could promote or inhibit the mitochondrial pathway of apoptotic cell death. As compound **1** induced apoptosis in 4T1 cells, in order to further reveal possible underlying apoptotic mechanisms, we assessed the effects of compound **1** on the expression of pro-apoptotic Bax and anti-apoptotic Bcl-2 in 4T1 cells by flow cytometry. As illustrated in Figure 1B, compound **1** treatment increased the percentage of Bax^+^4T1 cells compared to 4T1 cells. Further, a decrease in the percentage of Bcl-2^+^4T1 cells was observed after compound **1** treatment compared with control cells (Figure 1C). Further, the Bax/Bcl-2 ratio was calculated. Compound **1** treatment significantly increased the Bax/Bcl-2 ratio compared with 4T1 cells (Figure 1D), indicating a shift toward a pro-apoptotic response and potentially making 4T1 cells more prone to apoptosis. This imbalance between pro-apoptotic and anti-apoptotic proteins following compound **1** treatment could be one of the mechanisms involved in triggering 4T1 cell death. It is known that increased pro-apoptotic signaling can lead to the activation of executioner caspases [50]. As illustrated in Figure 1E, we examined changes in the percentage of caspase-3^+^4T1 cells after compound **1** treatment compared with nontreated cells using flow cytometry. Compound **1** treatment increased the percentage of caspase-3^+^4T1 cells compared with nontreated 4T1 cells; however, this increase did not reach statistical significance (Figure 1E). Bcl-6 is a transcriptional repressor and oncogenic driver involved in proliferation, differentiation, and apoptosis. In a study by Walker S. et al., Bcl-6 was recognized as a potential molecular target for breast cancer therapy, while Bcl-6 overexpression is linked to increased proliferation and survival of various breast cancer cell lines [51]. As illustrated in Figure 1F, compound **1** treatment decreased the percentage of Bcl-6^+^4T1 cells compared with 4T1 cells. This reduction may contribute to the pro-apoptotic effects of compound **1** in 4T1 cells. Taken together, these findings indicate that compound **1** induced apoptosis in 4T1 cells, which is accomplished by a shift in balance between pro-apoptotic and anti-apoptotic proteins towards a pro-apoptotic profile.

### 3.4. Effects of Compound ***1*** on 4T1 Cell Proliferation

**Figure 2:**
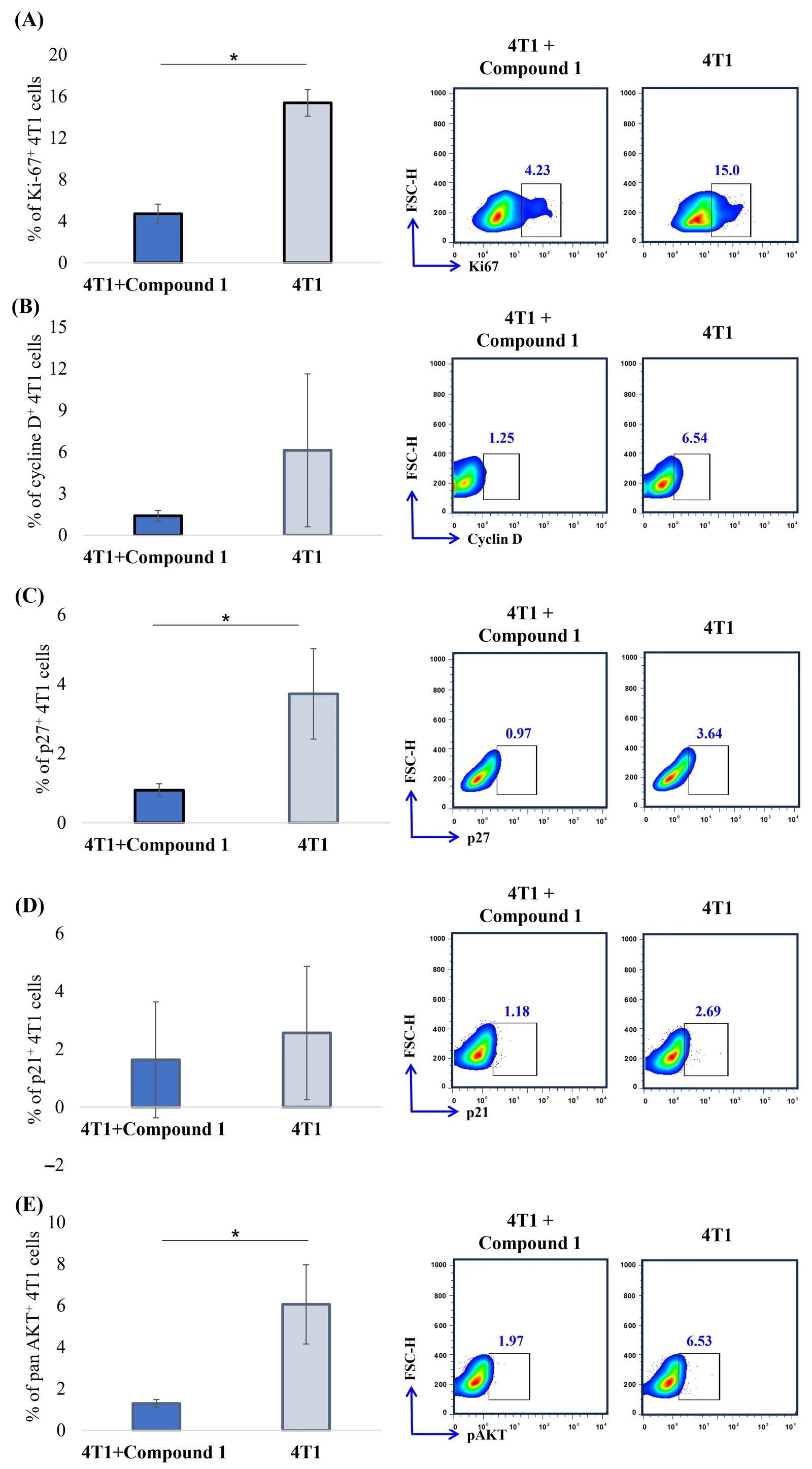
Compound **1** reduces the proliferation of 4T1 cells. Analysis of Ki-67 expression in 4T1 cells after compound **1** treatment (94 μM for 24 h), as well as control cells, was conducted by flow cytometry (**A**). Analysis of cyclin D3 (**B**), p27 (**C**), p21 (**D**), and panAKT (**E**) expression following Compound **1** treatment using flow cytometry. Representative dot plots are presented. Data are shown as means ± SDs of 3 independent experiments. Mann–Whitney U test: * *p* < 0.05 compared with the untreated group.

Cancer is characterized by the dysregulation of proteins involved in cell cycle progression, leading to uncontrolled cell proliferation [52]. Further, we investigated whether, besides apoptosis, compound **1** affects 4T1 cell proliferation. In order to determine the antiproliferative effects of compound **1**, the initial expression of Ki-67 in 4T1 cells were observed by flow cytometry. Ki-67 is a well-known proliferative marker, and its expression positively correlates with poor breast cancer prognosis, therapeutic response, and cancer aggressiveness [53]. As illustrated in Figure 2A, the percentage of Ki-67^+^4T1 cells was significantly lower following compound **1** treatment compared to the control 4T1 cells, indicating reduced proliferative capacity of 4T1 cells after compound **1** treatment. As Ki-67 is known to vary during cell cycle progression, we next examined the expression of Cyclin D, p21, and p27 [54]. Cyclin D, a key regulator of cell cycle progression, promotes the transition from the G1 to S phase of the cell cycle [55]. After compound **1** treatment, a decrease in cyclin D^+^4T1 cells was observed; however, this difference did not reach statistical significance (Figure 2B). Aside from cyclins, cyclin-dependent kinases (CDKs) and their inhibitors are essential for the regulation of cell cycle progression [56]. Among cyclin-dependent kinase inhibitors, p21 and p27 play important roles in progression from the G1 to S phase of the cell cycle by inhibiting cyclin-CDK complexes [57,58]. After compound **1** treatment, a significant decrease in the percentage of p27^+^4T1 cells was observed compared with the 4T1 cells (Figure 2C). Further, the percentage of p21^+^4T1 cells also decreased; however, this difference did not reach statistical significance (Figure 2D). These data suggested that compound **1** could modulate the expression of cell cycle regulatory proteins in 4T1 cells. All three AKT isoforms play essential roles in many fundamental cellular processes. AKT signaling is involved in the regulation of cell proliferation, cell cycle progression, and apoptosis [59]. Also, AKT represents a therapeutic target in breast cancer, and capivasertib, a pan-AKT inhibitor, has been approved for the treatment of specific molecular subtypes of advanced breast cancer [60]. Compound **1** treatment significantly decreased the percentage of pan-AKT^+^4T1 cells compared with 4T1 cells, as illustrated in Figure 2E. Taken together, these results indicate that compound **1** may reduce the proliferative capacity of 4T1 cells.

### 3.5. Immunomodulatory Effects of Compound ***1***

**Figure 3:**
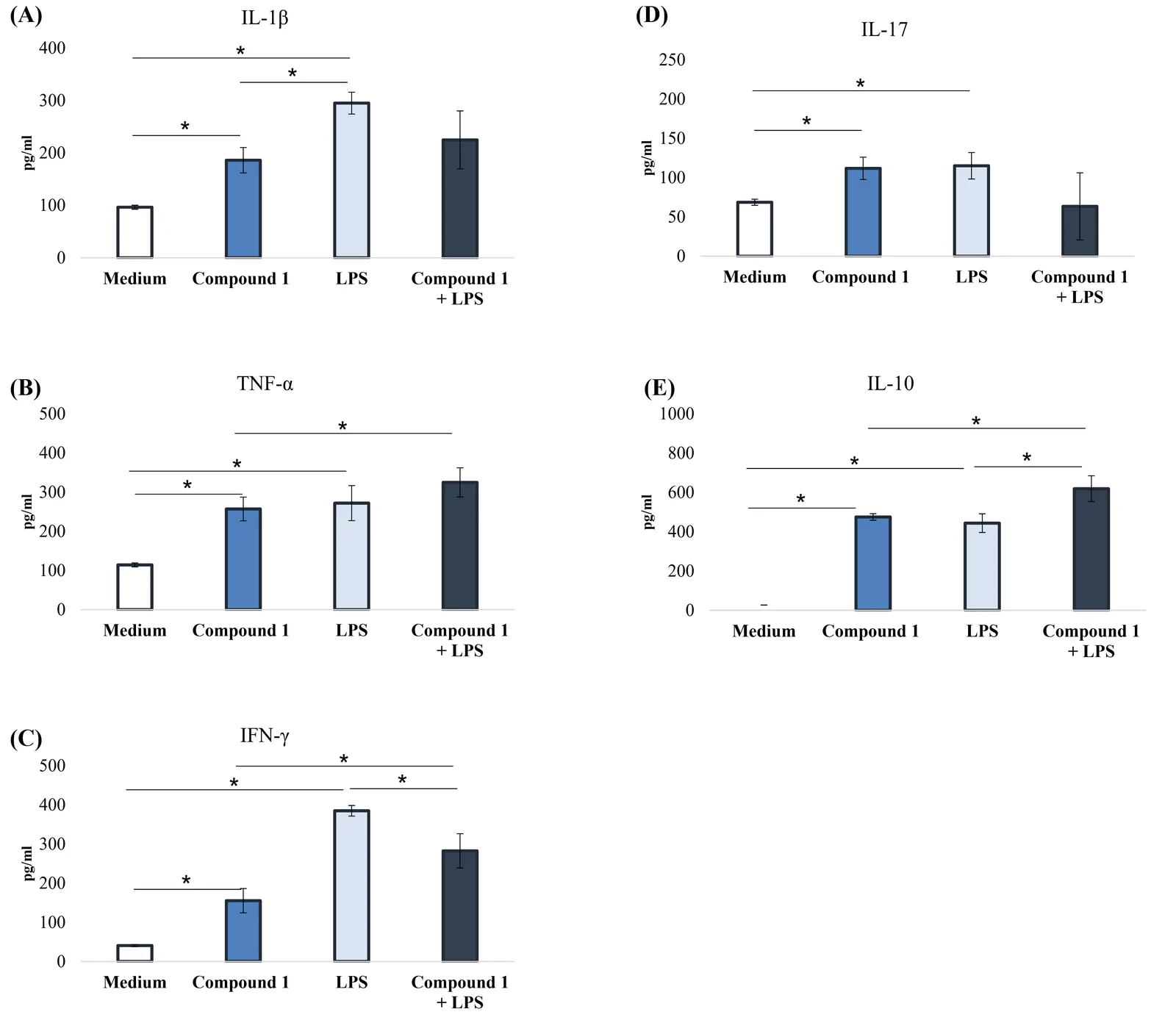
The splenocyte supernatant concentrations of IL-1β (**A**), TNF-α (**B**), IFN-γ (**C**), IL-17 (**D**), and IL-10 (**E**), measured after LPS stimulation or/and compound **1** treatment. ELISA was used to determine cytokine concentrations. Data are presented as means ± SDs of five mice per group and are representative of three separate experiments. Statistical significance was determined by the Kruskal–Wallis test, followed by the Mann–Whitney U test. * *p* < 0.05.

Our next objective was to assess the cytokine levels in splenocytes following treatment with LPS/ConA, compound **1**, or their co-treatment. LPS, also known as endotoxin, is a major component of the outer membrane of Gram-negative bacteria and is released during bacterial lysis [61]. Its toxic effects are primarily mediated by lipid A, which can induce a strong inflammatory response in the host [62]. LPS is widely used in studies of innate immunity because it activates immune cells through signaling pathways mediated by Toll-like receptor 4 (TLR4) [63]. Accordingly, in the present study, LPS was used to activate innate immune cells. As expected, stimulation with LPS significantly increased the production of all tested cytokines compared to the control cells cultured in medium (Figure 3). Treatment with compound **1** alone also increased the levels of all examined cytokines compared to control splenocytes, indicating that compound **1** may promote a pro-inflammatory cytokine profile in non-activated innate immune cells (Figure 3). Furthermore, co-treatment with compound **1** and LPS increased the concentration of TNF-α in comparison to LPS-stimulated cells; however, this effect did not reach statistical significance (Figure 3). In contrast, co-cultivation significantly reduced the concentration of pro-inflammatory IFN-γ, as well as IL-1β and IL-17 (this did not reach statistical significance) compared to LPS-treated cells (Figure 3). Additionally, simultaneous treatment with compound **1** and LPS statistically significantly increased the level of anti-inflammatory IL-10 (Figure 3). Taken together, these results suggest that compound **1** exerts a potential immunomodulatory effect, with a pronounced anti-inflammatory effect in LPS-activated innate immune cells in vitro, characterized by the suppression of pro-inflammatory cytokines and the enhancement of anti-inflammatory IL-10.

**Figure 4:**
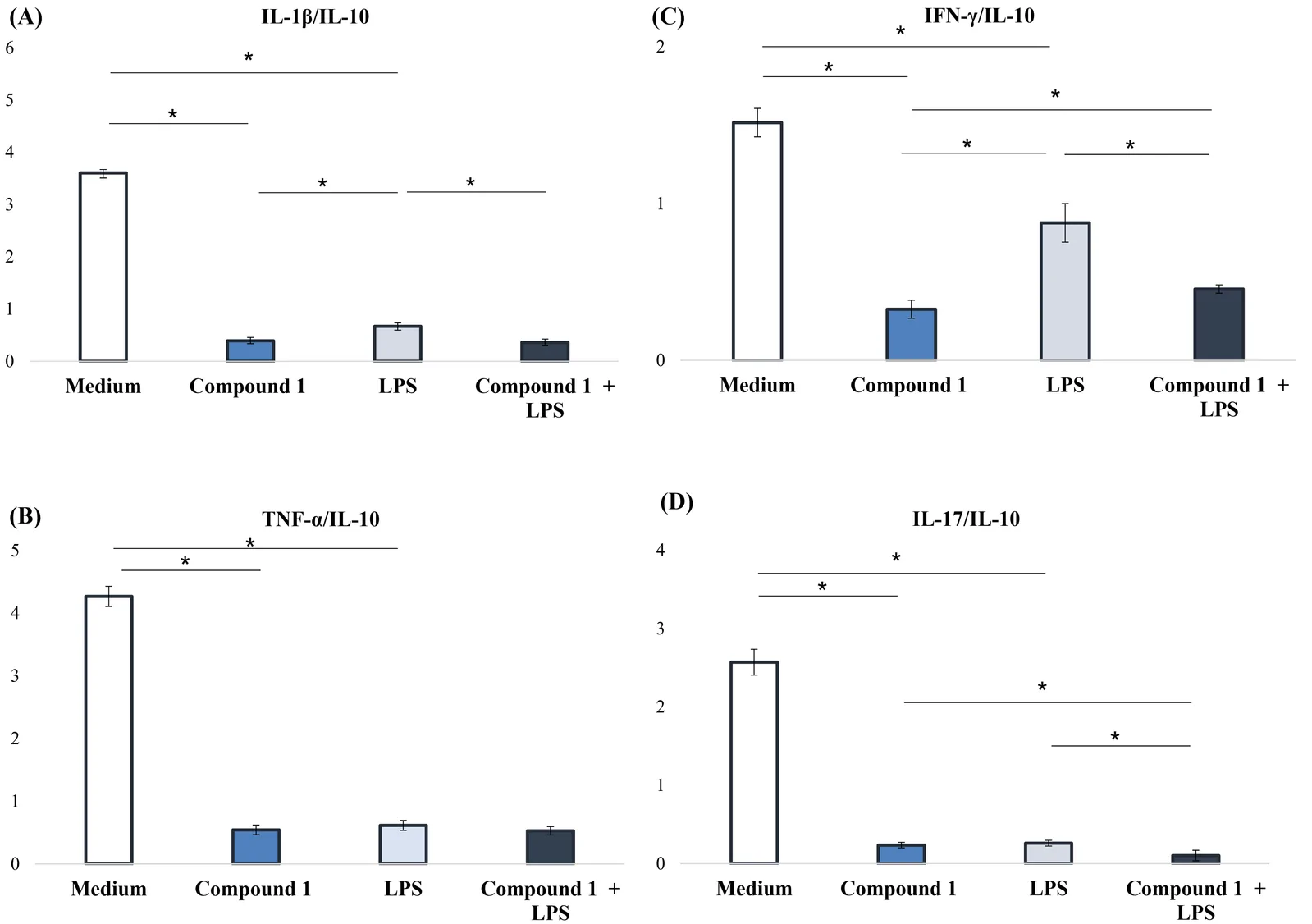
Ratio of pro- and anti-inflammatory cytokine concentrations produced by splenocytes incubated with medium only, compound **1,** LPS, or a combination of compound **1** and LPS. An ELISA was used to determine cytokine concentrations. Data are presented as means ± SDs of five mice per group and are representative of three separate experiments. Statistical significance was determined by the Kruskal–Wallis test, followed by the Mann–Whitney U test. * *p* < 0.05.

To further characterize this effect, the ratios between counter-regulatory cytokines were calculated to assess whether the resulting cytokine profile was predominantly pro-inflammatory or anti-inflammatory. LPS treatment resulted in a significant decrease in the ratios of TNF-α/IL-10, IL-1β/IL-10, IL-17/IL-10, and IFN-γ/IL-10 compared with untreated cells (Figure 4). Compound **1** also induced a significant reduction in all analyzed ratios of pro-inflammatory cytokines (TNF-α, IL-1β, IFN-γ, and IL-17) to the anti-inflammatory cytokine IL-10 compared with unstimulated splenocytes (Figure 4). Co-treatment with LPS and compound **1** significantly reduced the IL-1β/IL-10, IL-17/IL-10, and IFN-γ/IL-10 ratios compared with LPS-treated cells (Figure 4). Although, the TNF-α/IL-10 ratio also showed a decreasing trend, the difference did not reach statistical significance (Figure 4). These findings indicate that the immunomodulatory activity of compound **1** may depend on the activation status of immune cells by elevating cytokine levels in non-activated cells while attenuating inflammatory responses in already activated cells.

**Figure 5:**
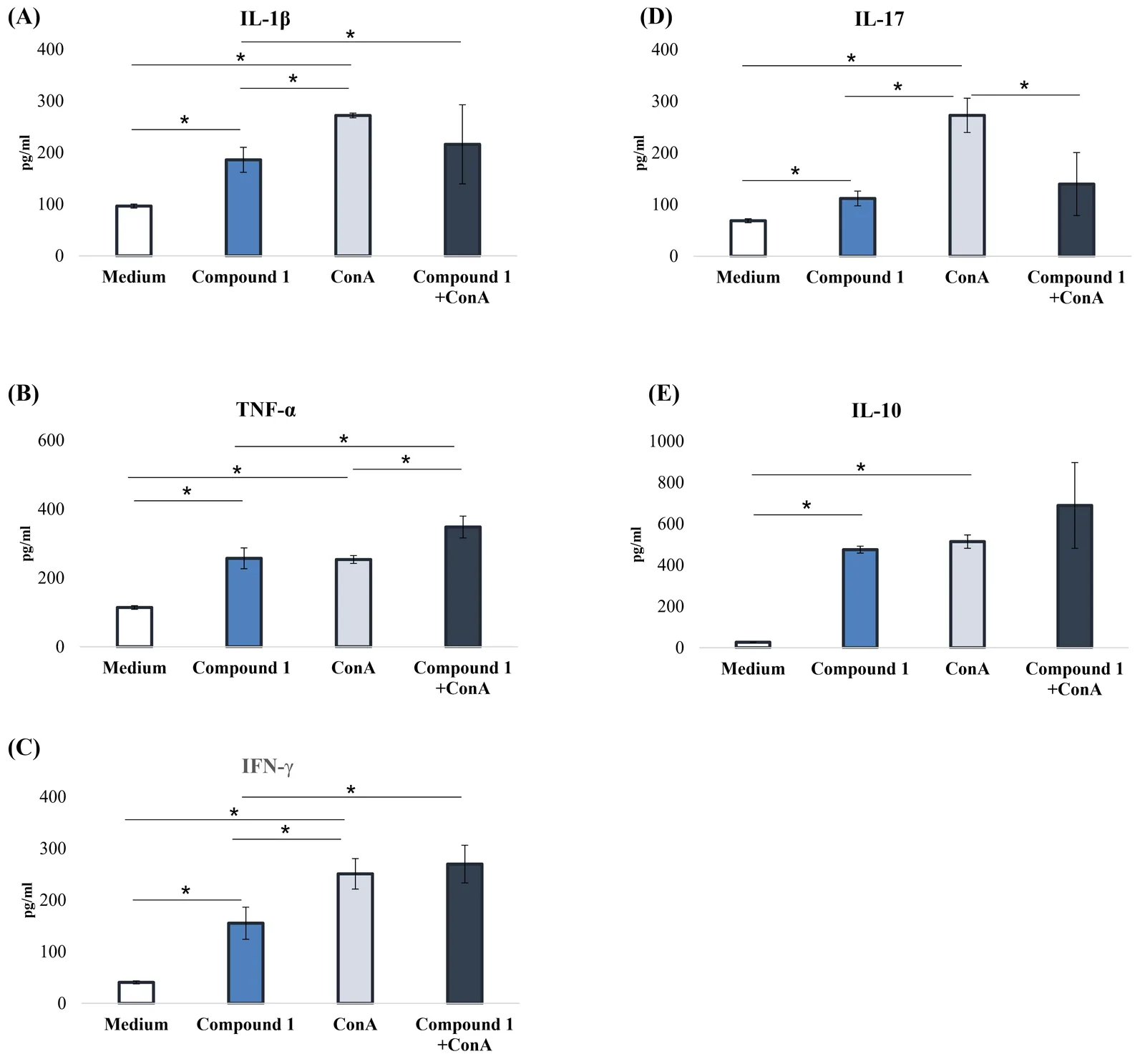
Concentrations of IL-1β (**A**), TNF-α (**B**), IFN-γ (**C**), IL-17 (**D**), and IL-10 (**E**) measured in the supernatant of splenocytes isolated from BALB/c mice after cultivation with medium, compound **1**, ConA, or co-cultivation with ConA and compound **1**. ELISA was used to determine cytokine concentrations. Data are presented as means ± SDs of five mice per group and are representative of three separate experiments. Statistical significance was determined by the Kruskal–Wallis test, followed by the Mann–Whitney U test. * *p* < 0.05.

ConA is a lectin that binds predominantly to carbohydrates containing mannose and glucose [64]. As a potent T-cell mitogen, ConA stimulates adaptive immune responses and promotes cytokine secretion [65,66]. Therefore, ConA was used as a stimulator of cells of the adaptive immune system. To determine whether compound **1** affects the functional capacity and immunomodulatory properties of splenocytes treated and untreated with ConA, the production of various cytokines (IL-1β, TNF-α, IFN-γ, IL-17, and IL-10) was assessed. Stimulation with ConA significantly increased concentrations of all tested cytokines compared to control cells, as anticipated (Figure 5). Furthermore, co-treatment with compound **1** and ConA significantly decreased the concentrations of IL-1β and IL-17, while increasing the concentration of anti-inflammatory IL-10; however, this increase did not reach statistical significance compared with the ConA stimulated group (Figure 5). In contrast, TNF-α concentration was significantly increased in the co-treated group, whereas IFN-γ also showed an increasing trend without statistical significance. Regarding the ratios of counter-regulatory cytokines, ConA significantly reduced all measured ratios of pro-inflammatory and anti-inflammatory cytokines compared to untreated cells (Figure 5).

**Figure 6:**
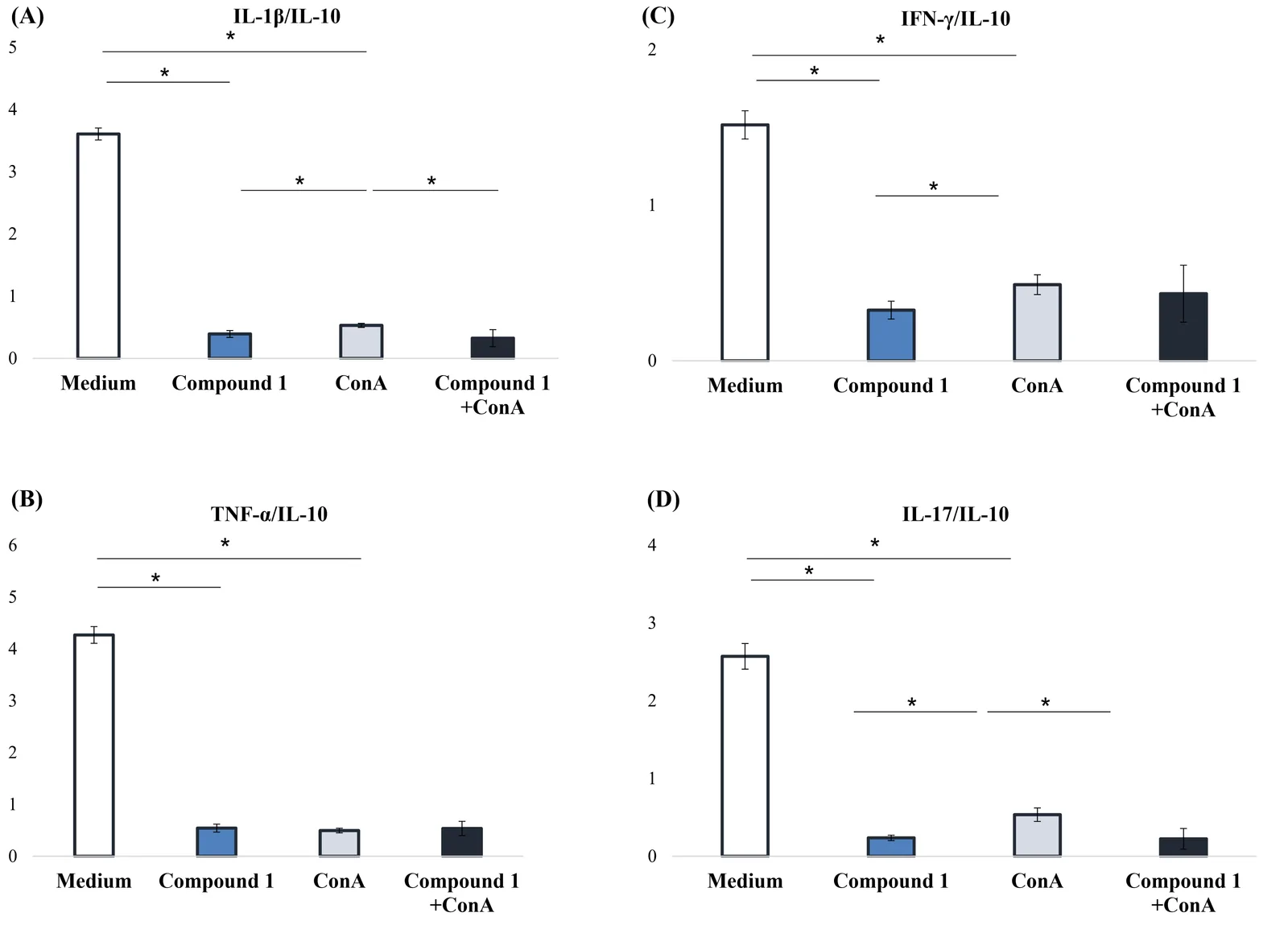
Ratios of pro- and anti-inflammatory cytokine concentrations produced by splenocytes incubated with medium only, compound **1,** ConA, or a combination of compound **1** and ConA. ELISA was used to determine cytokine concentrations. Data are presented as means ± SDs of five mice per group and are representative of three separate experiments. Statistical significance was determined by the Kruskal–Wallis test, followed by the Mann–Whitney U test. * *p* < 0.05.

Compound **1** also reduced the ratios of TNF-α/IL-10, IL-1β/IL-10, IL-17/IL-10, and IFN-γ/IL-10 compared to untreated cells (Figure 6). Co-treatment with compound **1** and ConA resulted in a decrease in the ratios of IL-1β/IL-10 and IL-17/IL-10 compared to ConA-treated cells, while the decrease in IFN-γ/IL-10 did not reach statistical significance. In contrast, the ratio of TNF-α/IL-10 was increased under the same conditions, but the increase did not reach statistical significance (Figure 6). Taken together, compound **1** alone may exhibit a pro-inflammatory effect on non-activated adaptive immune cells. However, when tested on activated cells of the adaptive immune response, compound **1** demonstrated an anti-inflammatory effect by significantly reducing the levels of pro-inflammatory IL-1β and IL-17, while, at the same time, elevating the levels of anti-inflammatory cytokine IL-10.

Our findings are consistent with previous studies showing that COX inhibitors are able to modulate cytokine production and attenuate inflammatory responses. Several novel pyrazole- and tetrazole-based COX-2 inhibitors have been shown to suppress the expression of pro-inflammatory mediators, particularly TNF-α and IL-6, while simultaneously exhibiting potent anti-inflammatory activity comparable to or even greater than that of celecoxib, a clinically established selective COX-2 inhibitor [67,68]. Similarly, the selective COX-2 inhibitor, etoricoxib, reduced elevated levels of pro-inflammatory cytokines IL-1β, TNF-α, and IFN-γ and restored expression of anti-inflammatory cytokines in a rat model of lung cancer induced by DMBA [69].

Overall, the present results indicate that tested compound **1** has the ability to modulate immune responses by suppressing the production of pro-inflammatory cytokines while promoting anti-inflammatory cytokine production. Therefore, compound **1** may represent a promising anti-inflammatory agent for diseases mediated by activated immune cells.

### 3.6. In Vitro COX Inhibition Assay

The in vitro inhibitory potential of the synthesized compounds was evaluated using commercial fluorometric assay kits, with oxaprozin serving as the parent compound for comparison. Compounds that showed more than 50% inhibition of the enzyme at a concentration of 100 μM were selected to determine the IC_50_ value. The IC_50_ value was defined as the concentration required to achieve a 50% reduction in enzyme activity. The results of the COX inhibition assays are summarized in Table 4. As shown, the synthesized oxaprozin derivatives showed moderate affinity for COX-1, and their percentage enzyme inhibition varied significantly within the series, highlighting the impact of structural modifications on the interaction with the enzyme. The minimal difference in the structure of compounds **1** and **2** also causes a significant difference in inhibitory activity. Namely, the derivative containing β-alanine in the side chain exhibited more than 50% inhibition of COX-1 at a concentration of 100 μM, indicating moderate inhibitory activity toward this enzyme. However, the chain extension of compounds **3** and **5** does not lead to retention of the inhibitory activity but to a drastic reduction, which definitely indicates the necessity of an optimal chain length. The significantly higher COX-1 inhibitory activity of compound **4**, compared to other derivatives, indicates that the presence of a tryptophan residue in the side chain contributes considerably to the observed effect. This indicates that the indole ring in the side chain of tryptophan contributes π-electrons in various interactions and participates in its optimal fit within the active site of the enzyme. Although oxaprozin belongs to the group of non-selective inhibitors, some studies indicate that it shows a slightly higher affinity for COX-1, which is confirmed by the results of our in vitro COX inhibition assay. Unfortunately, the results indicate that no newly synthesized compound achieved more than 50% inhibition of the enzyme at a concentration of 100 μM.

### 3.7. Molecular Docking Simulation Studies

A molecular docking study was carried out to better understand the binding patterns of the tested compounds within the active sites of the COX-1 and COX-2 enzymes. The docking protocol was subsequently validated by redocking the co-crystallized ligands, and its reliability was assessed based on the resulting RMSD values. The calculated values were less than 2 Å (5WBE: 0.4877 Å, and 1CX2: 1.2460 Å), confirming the predicted binding mode. The binding potential was estimated based on the number and type of non-covalent contacts with significant interacting residues, as well as the docking scores.

Although the sequence homology between the two cyclooxygenase isoforms is only 65%, the structural differences in the catalytic sites are minimal. The development of selective COX-2 inhibitors is based on these differences. The main difference is the existence of an additional pocket within the active site, which is more accessible in the COX-2 isoform, as the amino acid residue Ile523 in COX-1 has been replaced by the less bulky side chain residue Val523. The large volume of the isoleucine residue in the side chain takes up space in the cavity of the active site of COX-1, while the substituted valine in COX-2 leads to conformational changes in Tyr355, and thus opens an additional pocket. In addition, a modification at position 434, such as replacing isoleucine with valine, contributes to the larger active site of COX-2 by displacing the side chain Phe518. This additional pocket enables interactions with Arg513, which is substituted for His513 in COX-1. The existence of these three structural differences allows the inhibitors to access a pocket on the side of the catalytic site of COX-2 that is not readily accessible in COX-1.

**Figure 7:**
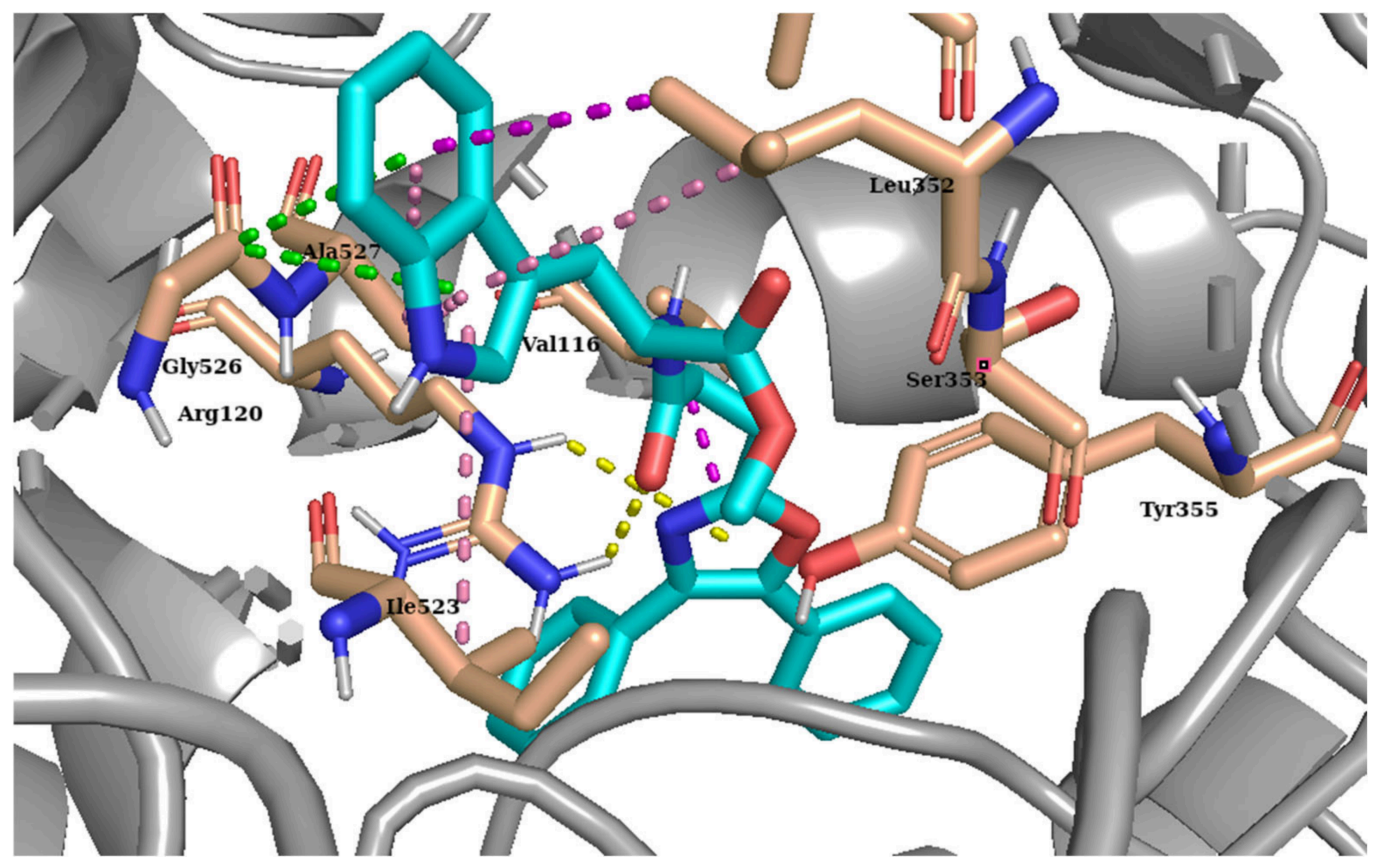
Predicted binding mode of compound **4** within the active site of COX-1 (PDB ID: 5WBE). Three-dimensional molecular docking model; non-covalent interactions are represented by dashed lines: conventional hydrogen bonds (yellow), π-alkyl contacts (pink), π-σ (magenta), and amide-π-stacked (green).

The results of a molecular docking study demonstrate that oxaprozin binds to the active site of COX-1, with a binding energy of −8.7 kcal/mol, which does not represent the most stable complex formed with this enzyme. The carbocyclic aromatic systems of this compound are surrounded by the amino acid residues Val116, Val349, Leu352, and Ala527, which form hydrophobic interactions of the π-alkyl and π-ơ types, while the oxazole ring establishes a π-ơ interaction with Ile523. Compound **1** forms additional hydrophobic interactions, including a π-alkyl interaction with Leu531 and a π-π T-shaped interaction with Tyr355. In addition, a carbon-hydrogen bond with Met522 was observed. A similar binding pattern to the COX-1 active site was identified for compounds **2**, **3**, and **5**, which was further reflected in their comparable docking scores. In these derivatives, conventional hydrogen bonding interactions occur with the key active site amino acid Arg120 (between the carbonyl groups within the side chain). As presented in Table 5, compound **4** established the highest number of key hydrophobic interactions within the COX-1 enzyme’s active-site gorge (Figure 7). These contacts were formed with residues Val116, Val349, Leu352, Ile523, Gly526, and Ala527, while Ser353, Tyr355, and Val349 established van der Waals forces with compound **4**. These findings confirm the results of the in vitro COX inhibition assay and the significantly higher COX-1 inhibitory activity of compound **4** compared to all tested derivatives, even the parent compound.

The COX-2 enzyme has a predominantly hydrophobic active site in which the inhibitors mainly achieve van der Waals forces. According to the molecular docking results, oxaprozin binds within the COX-2 active site where its two phenyl rings occupy a hydrophobic surface formed by amino acid residues Val349, Leu352, Ala527, and Leu531. In addition, the aliphatic side chain of Val523 interacts with the oxazole core of oxaprozin and forms a hydrophobic π-σ interaction. The carboxyl group forms three conventional hydrogen bonds with Leu352, Ser353, and His90 residues, thereby contributing to the stability of the drug–receptor complex. Compared to SC-558, the phenyl ring of oxaprozin achieves only one significant electrostatic interaction with Arg120, while the hydrogen bond with Arg513 and the water bridges formed with the backbone of Phe518 are absent. Molecular dynamics simulation results by Soliva et al. indicate that four important residues (Arg120, Asn192, Leu352, and Arg513) form significant electrostatic interactions with SC-558, while other residues establish van der Waals forces [70].

The obtained molecular docking results indicated that the newly synthesized compounds are anchored within the pocket formed by amino acid residues Val349, Leu352, Val523, Ala527, and Leu531 in the COX-2 binding cavity, where they predominantly form hydrophobic interactions. All tested oxaprozin derivatives establish interactions with these residues, except for compound **4**. Compounds **2** and **3** showed the same mode of binding interaction, which explains their similar docking score values. However, the absence of crucial electrostatic binding interactions led to the creation of differences in geometry and a decrease in binding affinity within the active site of COX-2, although these compounds exhibited the most favorable docking scores (−9.8 and −9.9 kcal/mol). The mentioned compounds bind to the active site of the target enzyme via two conventional hydrogen bonds formed between hydrogen atoms of their amide groups and the carbonyl oxygen of amino acid residues Leu352 and Ser353 (2.83 and 2.24 for compound **2**; 2.81 and 2.40 Å for compound **3**). Compound **4** deviates from the standard binding pattern observed in other oxaprozin derivatives, which can be partially explained by its very bulky structure. Namely, in addition to the oxaprozin backbone, this compound also contains a tryptophan moiety that probably cannot be incorporated into the cavity of the active site. Also, the amino acid residue Tyr115 formed an unfavorable donor–donor non-bonding interaction with compound **4**, which further affects the increase in the energy of the system and significantly disrupts the stability of the compound–enzyme complex. This was also confirmed by the fact that compound **4** showed the least favorable docking score (−8.6 kcal/mol). Crystallographic data indicate the crucial importance of Tyr385, which donates a hydrogen atom to the heme that initiates activation of the enzyme [71]. Therefore, the notable exception to the interaction with this residue further supports the weak binding potential of the oxaprozin derivatives to COX-2.

## 4. Conclusions

In conclusion, this study demonstrates that structural modifications of oxaprozin significantly modulate its cyclooxygenase selectivity, cytotoxic potential, and immunomodulatory effects. While the newly synthesized derivatives show moderate affinity for COX-1 and limited inhibition of COX-2, compound **1** was identified as the most promising candidate due to its favorable selectivity index, demonstrating approximately twofold higher cytotoxicity toward cancer cells compared to non-cancerous mMSC and MRC-5 cells. Compound **1** can effectively target tumor cells, inducing potent apoptosis in 4T1 breast cancer cells through a strategic shift in the Bax/Bcl-2 balance and the downregulation of the oncogenic driver Bcl-6. Furthermore, its potent antiproliferative activity was demonstrated by a significant reduction in Ki-67 expression and the suppression of the pan-AKT signaling pathway. Overall, these findings indicate that compound **1** has the ability to modulate immune responses by suppressing the production of pro-inflammatory cytokines while promoting anti-inflammatory cytokine production under stimulated conditions. Its potent antitumor activity may not be directly related to cyclooxygenase inhibition, suggesting the involvement of COX-independent pathways. Therefore, compound **1** may represent a promising anti-inflammatory agent for the treatment of diseases mediated by activated immune cells.

## Figures and Tables

**Table 1:** Target protein data.

Target Protein	Selected PDB (Resolution)	Organism	Co-Crystallized Ligand	Chains	Selected Chain
**COX-1**	5WBE (2.75 Å)	Ovis aries	Mofezolac 2-[3,4-bis(4-methoxyphenyl)-1,2-oxazol-5-yl] acetic acid	A, B	A
**COX-2**	1CX2 (3.00 Å)	Mus musculus	SC-558 (1-phenylsulfonamide-3-trifluoromethyl-5-parabromophenylpyrazole)	A, B, C, D	A

**Table 2:** IC_50_ values of oxaprozin-based amide derivatives in different cell lines after 48 h of treatment.

	IC_50_ (μM)					
Cell Line	Compound 1	Compound 2	Compound 3	Compound 4	Compound 5	Oxaprozin
4T1	94.06 ± 14.19	91.17 ± 5.68	105.42 ± 14.32	88.73 ± 10.47	95.73 ± 5.49	76.33 ± 7.56
CT26	115.21 ± 11.67	123.03 ± 13.65	127.52 ± 17.61	101.13 ± 2.07	100.48 ± 16.49	88.76 ± 8.54
MCF-7	57.51 ± 9.62	48.75 ± 11.06	62.3 ± 14.01	71.96 ± 2.56	96.23 ± 6.23	90.04 ± 12.57
HCT116	121.09 ± 18.025	146.6 ± 8.64	160.03 ± 13.63	79.25 ± 9.46	104.38 ± 13.05	111.91 ± 9.49
mMSC	267.36 ± 12.09	97.81 ± 10.86	116.54 ± 13.24	77.72 ± 12.93	94.30 ± 13.29	109.69 ± 17.59
MRC-5	125.03 ± 13.84	94.47 ± 7.43	103.19 ± 13.28	101.04 ± 10.92	104.21 ± 13.21	66.01 ± 9.07

Data presented as means ± SDs.

**Table 3:** Index of selectivity (IC_50_ mMSC or MRC-5/IC_50_ tumor cells) of oxaprozin-based amide derivatives.

Cell Line	Compound 1	Compound 2	Compound 3	Compound 4	Compound 5	Oxaprozin
4T1	2.842	1.073	1.105	0.876	0.985	1.947
CT26	2.321	0.795	0.914	0.769	0.938	1.236
MCF-7	2.174	1.938	1.656	1.404	1.083	0.733
HCT116	1.033	0.644	0.645	1.275	0.998	0.590

**Table 4:** Inhibitory activity of oxaprozin derivatives on COX-1 and COX-2.

Compound	COX-1		COX-2	
	% of Inhibition (Conc. 100 μM)	IC_50_ μM	% of Inhibition (Conc. 100 μM)	IC_50_ μM
**1**	27.72	>100	26.80	>100
**2**	59.59	86.35 ± 14.41	20.59	>100
**3**	28.06	>100	7.10	>100
**4**	67.60	6.25 ± 0.41	17.04	>100
**5**	25.42	>100	31.59	>100
**Oxaprozin**	75.67	56.92 ± 1.80	32.24	>100

IC_50_ values are presented as means ± standard deviations.

**Table 5:** Molecular docking parameters of the tested compounds.

Compound	Target	Interacting Residue	Docking Score (kcal/mol)
**1**	COX-1	Val116 (π-σ), Val349 (π-σ), Leu352 (π-alkyl), Tyr355 (π-π T-shaped), Met522 (CHB), Ile523 (π-σ), Ala527 (π-σ), Leu531(π-alkyl)	−8.6
	COX-2	Arg120 (π-cation), Val349 (π-alkyl), Leu352 (π-alkyl), Ser353 (HB), Arg513 (CHB), Val523 (π-σ, π-alkyl), Leu531 (π-alkyl)	−8.9
**2**	COX-1	Arg120 (HB x2), Val349 (π-alkyl), Leu352 (π-σ, π-alkyl), Ser353 (π-σ), Ile523 (π-alkyl), Gly526 (amide-π-stacked), Ala527 (π-σ, π-alkyl)	−8.9
	COX-2	Arg120 (π-cation), Val349 (π-alkyl x2), Leu352 (π-alkyl, HB), Ser353 (HB), Leu359 (π-alkyl), Arg513 (CHB), Val523 (π-σ), Leu531 (π-alkyl)	−9.8
**3**	COX-1	Val116 (CHB), Arg120 (HB x2), Val349 (π-alkyl), Leu352 (π-σ, π-alkyl), Ser353 (π-σ), Ile523 (π-alkyl), Gly526 (amide-π-stacked), Ala527 (π-σ, π-alkyl)	−8.8
	COX-2	Arg120 (π-cation), Val349 (π-alkyl x2), Leu352 (π-alkyl, HB), Ser353 (HB), Leu359 (π-alkyl), Arg513 (CHB), Val523 (π-σ), Leu531 (π-alkyl)	−9.9
**4**	COX-1	Val116 (π-σ), Arg120 (HB x2), Val349 (vdW), Leu352 (π-σ, π-alkyl), Ser353 (vdW), Tyr355 (vdW), Ile523 (π-alkyl), Gly526 (amide-π-stacked), Ala527 (π-alkyl x2)	−10.0
	COX-2	Tyr115 (bump), Arg120 (π-alkyl)	−8.6
**5**	COX-1	Arg120 (HB x2), Val349 (π-alkyl), Leu352 (π-σ, π-alkyl), Ser353 (π-σ), Tyr355 (vdW), Ile523 (π-alkyl), Gly526 (amide-π-stacked), Ala527 (π-σ, π-alkyl)	−8.7
	COX-2	Arg120 (π-cation), Val349 (π-alkyl), Leu352 (π-alkyl, HB), Ser353 (HB), Arg513 (HB), Val523 (π-alkyl), Leu531 (π-alkyl)	−9.4
**Oxaprozin**	COX-1	Val116 (π-alkyl), Val349 (π-alkyl), Leu352 (π-σ), Ile523 (π-σ), Ala527 (π-alkyl x2)	−8.7
	COX-2	His90 (HB), Arg120 (π-cation), Val349 (π-alkyl x2), Leu352 (π-alkyl, HB), Ser353 (HB), Val523 (π-σ), Leu531 (π-alkyl)	−9.6

## Data Availability

The raw data supporting the conclusions of this article will be made available by the authors on reasonable request.

## Supplementary Materials

The following supporting information can be downloaded at https://www.mdpi.com/article/10.3390/pharmaceutics18101271/s1, Figure S1. Cytotoxicity of oxaprozin-based amide derivatives. Effects of compounds **1**–**5** on the viability of 4T1 (A), CT26 (B), mMSC (C), MCF-7 (D), HCT116 (E), and MRC-5 (F) cells after a 48 h period of incubation, analyzed with the MTT assay. All data are presented as mean values ± SDs from three independent experiments (each experiment was performed in triplicate). Figure S2. FT-IR spectrum of compound **1**. Figure S3. FT-IR spectrum of compound **2**. Figure S4. FT-IR spectrum of compound **3**. Figure S5. FT-IR spectrum of compound **4**. Figure S6. FT-IR spectrum of compound **5**. Figure S7. ^1^H NMR spectrum (a) and ^13^C NMR spectrum (b) of compound 1. Figure S8. ^1^H NMR spectrum (a) and ^13^C NMR spectrum (b) of compound **2**. Figure S9. ^1^H NMR spectrum (a) and ^13^C NMR spectrum (b) of compound **3**. Figure S10. ^1^H NMR spectrum (a) and ^13^C NMR spectrum (b) of compound **4**. Figure S11. ^1^H NMR spectrum (a) and ^13^C NMR spectrum (b) of compound **5**. Figure S12. HRMS spectrum of compound **1**. Figure S13. HRMS spectrum of compound **2**. Figure S14. HRMS spectrum of compound **3**. Figure S15. HRMS spectrum of compound **4**. Figure S16. HRMS spectrum of compound **5**.
