# Supplementary material for: Oxaprozin-Based Amide Derivatives as Multifunctional Agents: Synthesis, Characterization, and Comprehensive Biological Profiling
Source: Pharmaceutics. 2026 Oct 7;18(10):1271. doi: 10.3390/pharmaceutics18101271 (PMC13708156; doi:10.3390/pharmaceutics18101271)
Supplement: Supplementary file 1 [file pharmaceutics-18-01271-s001.zip › pharmaceutics-4593047-supplementary.pdf]

**Contents**

**Figure S1.** Cytotoxicity of oxaprozin-based amide derivatives. Effects of compounds **1-5** on the viability of 4T1 (A), CT26 (B), mMSC (C), MCF-7 (D), HCT116 (E), and MRC-5 (F) cells after a 48 h period of incubation analyzed with the MTT assay. All data are presented as mean values ± SDs from three independent experiments (each experiment was performed in triplicate). 3

**Figure S2.** FT-IR spectrum of compound **1**. 4

**Figure S3**. FT-IR spectrum of compound **2**. 4

**Figure S4**. FT-IR spectrum of compound **3**. 5

**Figure S5**. FT-IR spectrum of compound **4**. 5

**Figure S6**. FT-IR spectrum of compound **5**. 6

**Figure S7.** $^{1}H$ NMR spectrum (a) and $^{13}C$ NMR spectrum (b) of compound **1**. 7

**Figure S8.** $^{1}H$ NMR spectrum (a) and $^{13}C$ NMR spectrum (b) of compound **2**. 8

**Figure S9.** $^{1}H$ NMR spectrum (a) and $^{13}C$ NMR spectrum (b) of compound **3.** 9

**Figure S10.** $^{1}H$ NMR spectrum (a) and $^{13}C$ NMR spectrum (b) of compound **4**. 10

**Figure S11.** $^{1}H$ NMR spectrum (a) and $^{13}C$ NMR spectrum (b) of compound **5**. 11

**Figure S12**. HRMS spectrum of compound **1**. 12

**Figure S13**. HRMS spectrum of compound **2**. 13

**Figure S14**. HRMS spectrum of compound **3**. 14

**Figure S15**. HRMS spectrum of compound **4**. 15

**Figure S16**. HRMS spectrum of compound **5**. 16

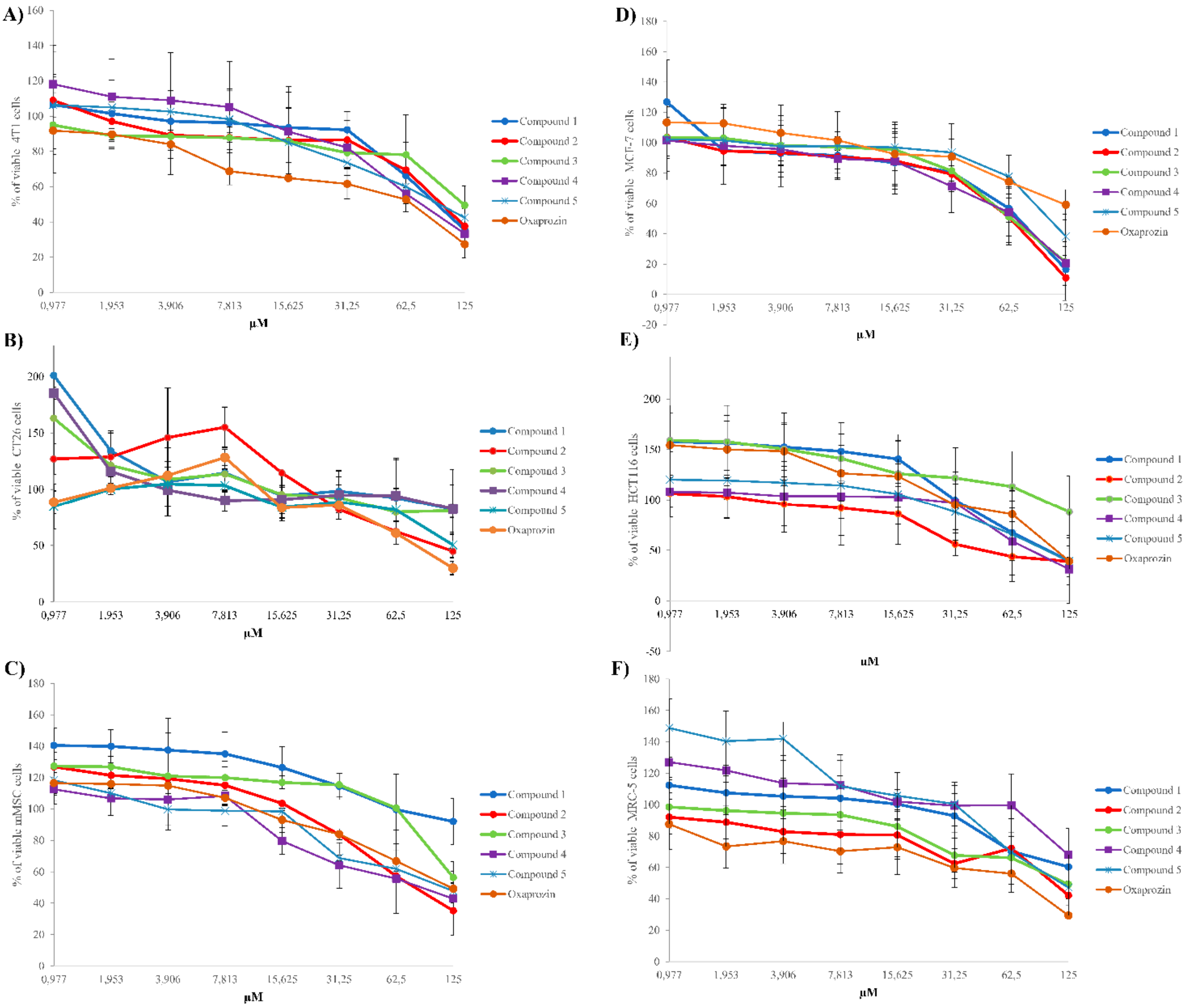


**Figure S1**. Cytotoxicity of oxaprozin-based amide derivatives. Effects of compounds **1-5** on the viability of 4T1 (A), CT26 (B), mMSC (C), MCF-7 (D), HCT116 (E), and MRC-5 (F) cells after a 48 h period of incubation analyzed with the MTT assay. All data are presented as mean values ± SDs from three independent experiments (each experiment was performed in triplicate).

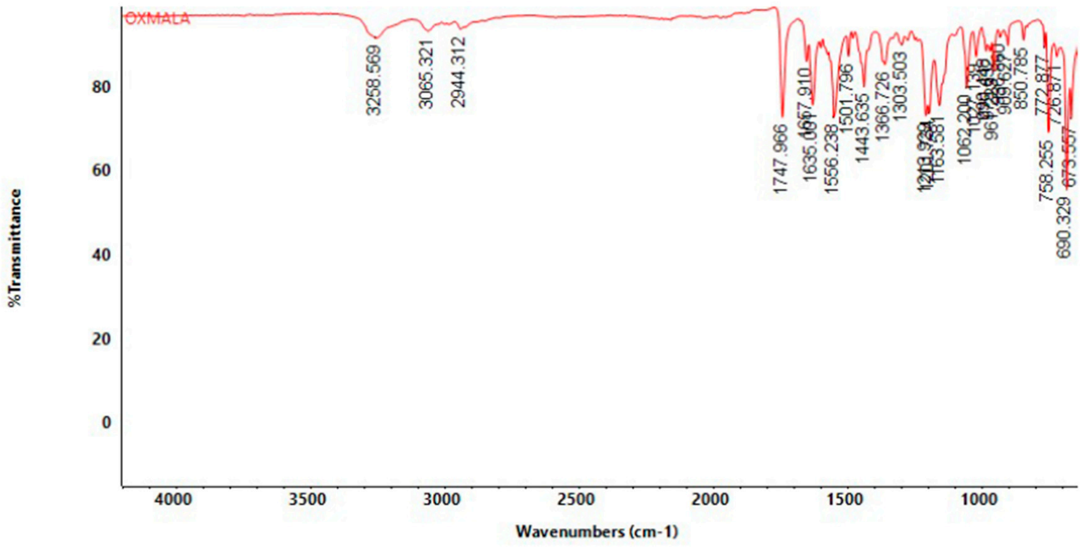


**Figure S2**. FT-IR spectrum of compound **1**.

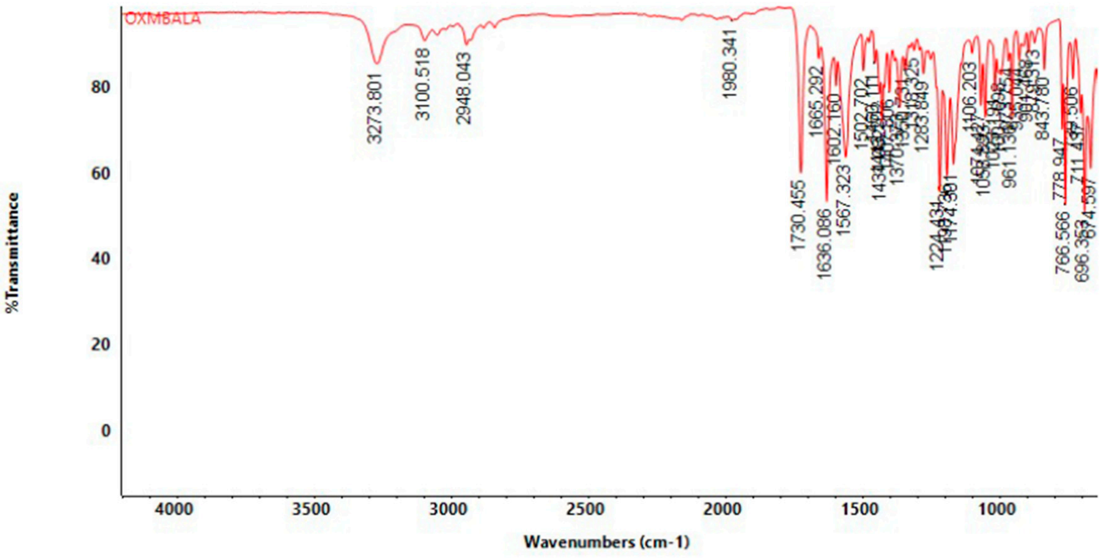


**Figure S3**. FT-IR spectrum of compound **2**.

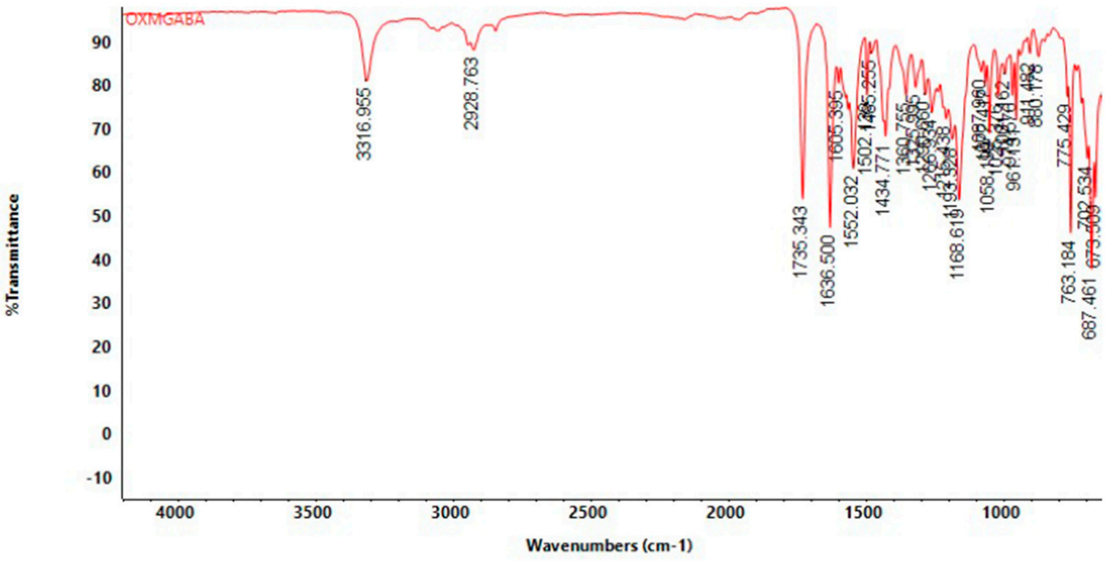


**Figure S4**. FT-IR spectrum of compound **3**.

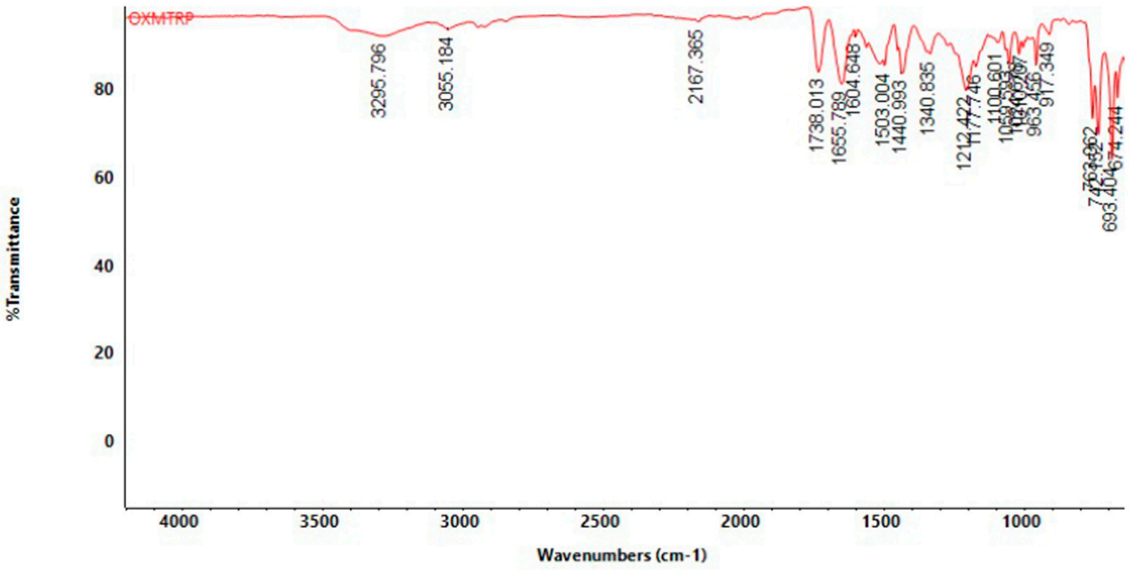


**Figure S5**. FT-IR spectrum of compound **4**.

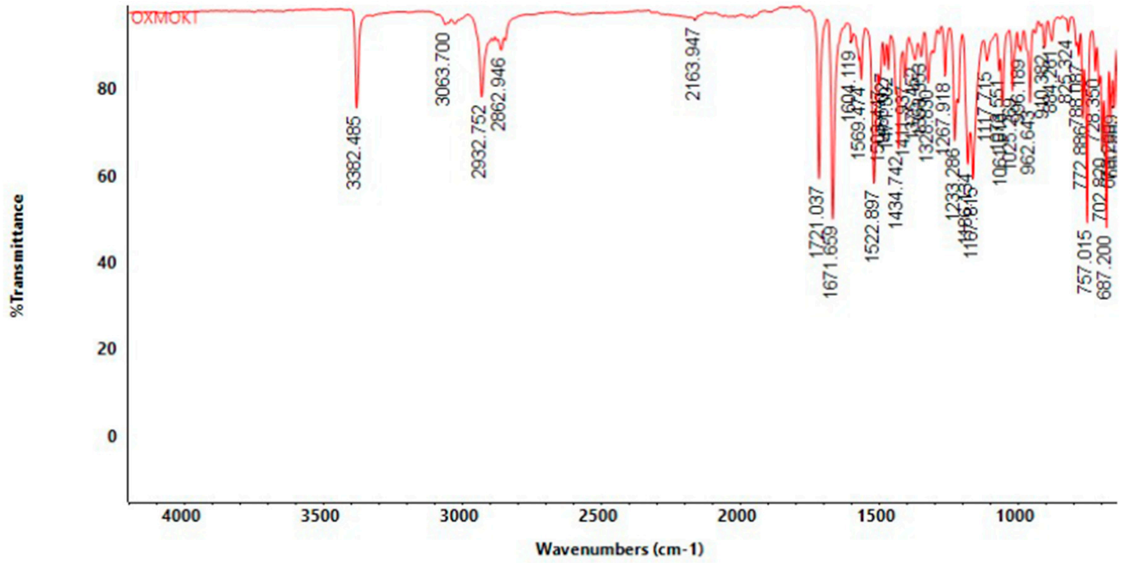


**Figure S6**. FT-IR spectrum of compound **5**.

a)

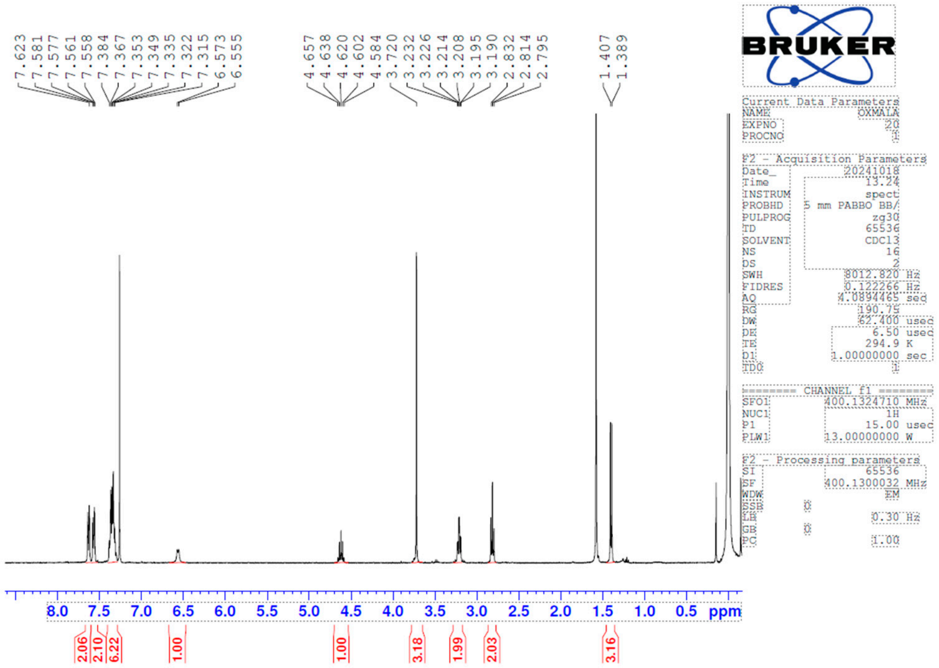

b)

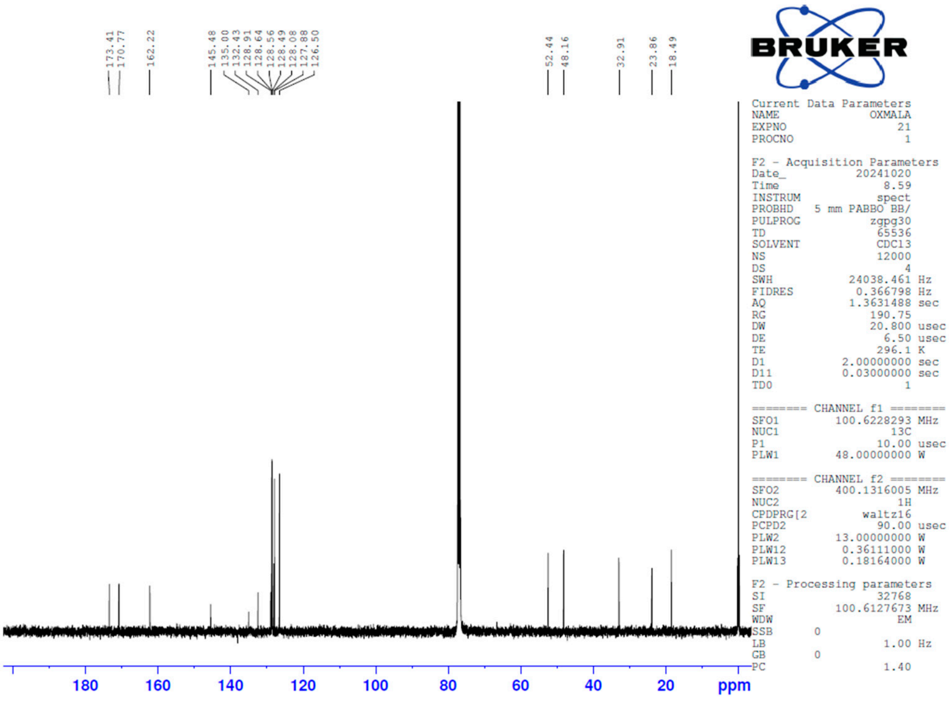

**Figure S7**. $^{1}H$ NMR spectrum (a) and $^{13}C$ NMR spectrum (b) of compound **1**.

a)

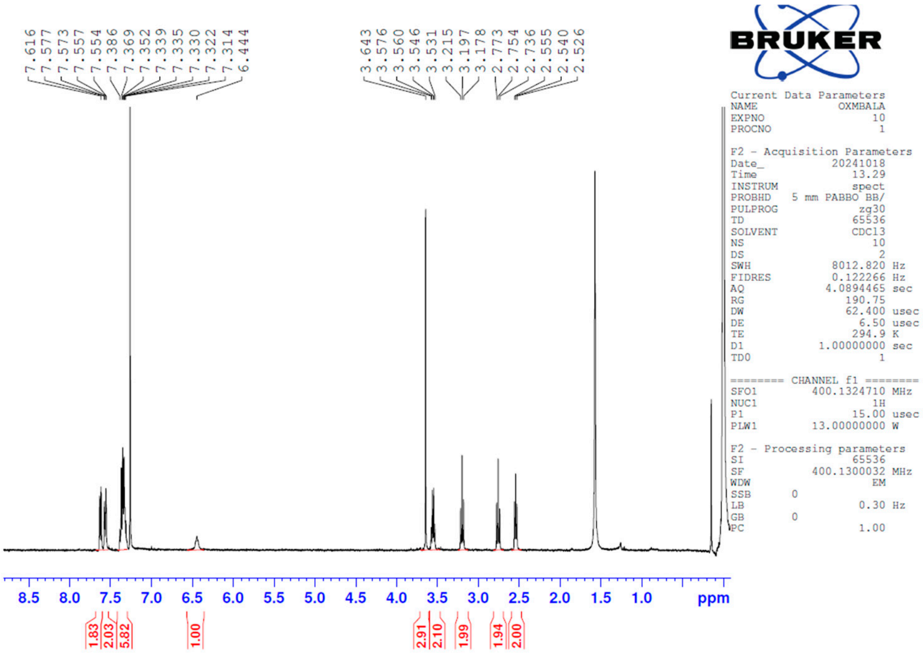

b)

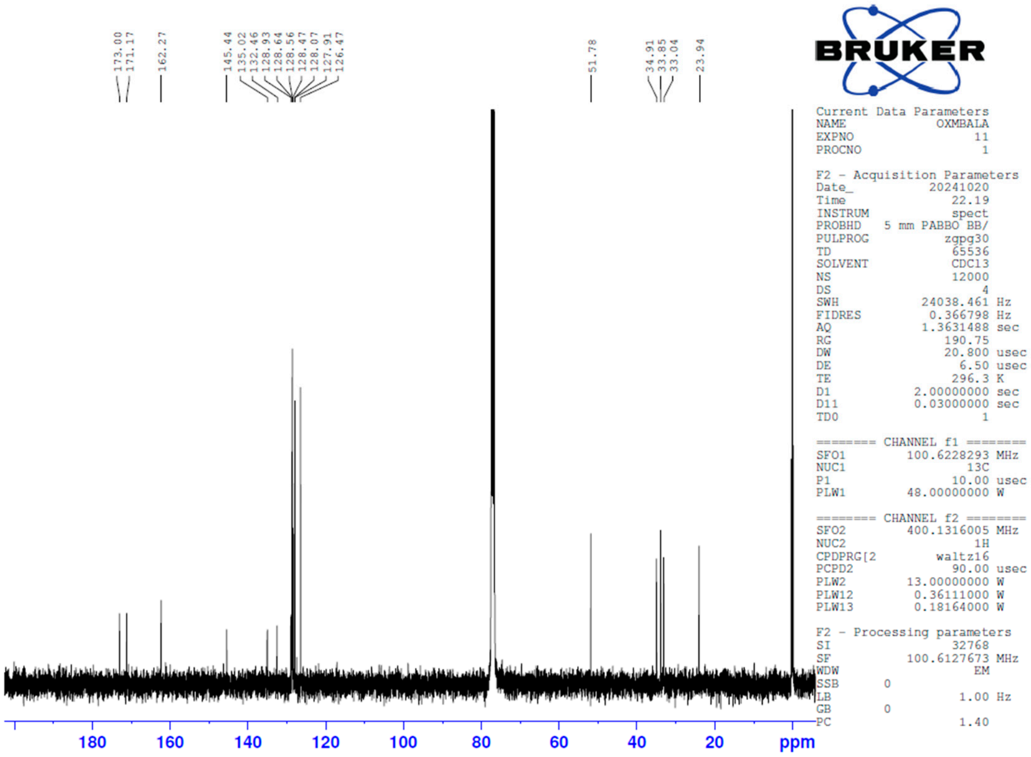

**Figure S8**. $^{1}$H NMR spectrum (a) and $^{13}$C NMR spectrum (b) of compound **2**.

a)

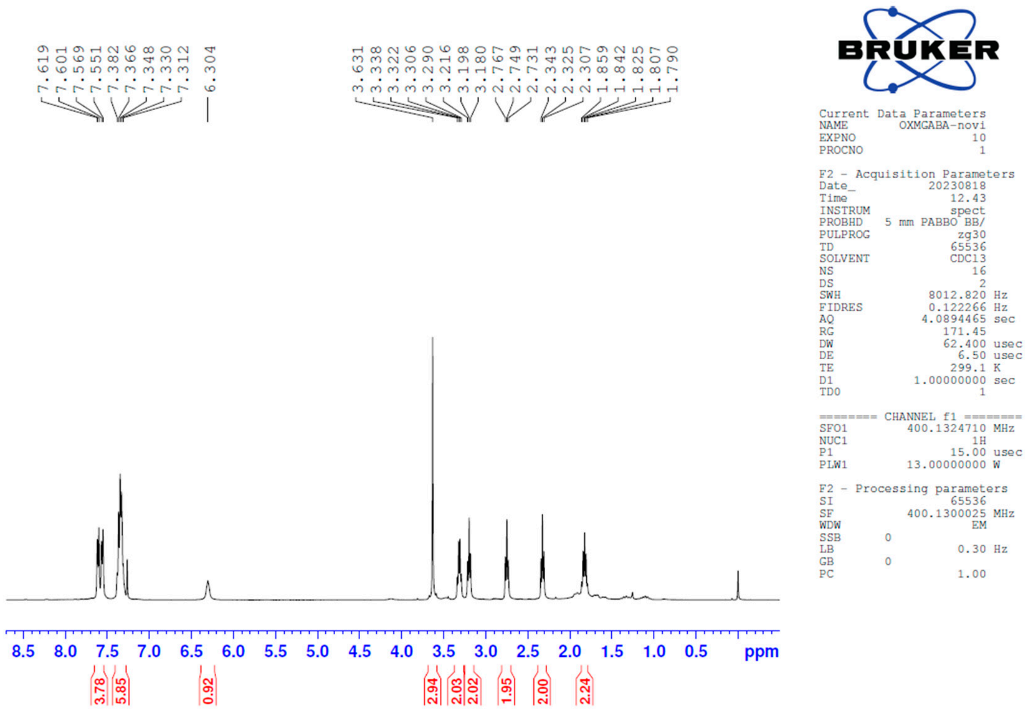


b)

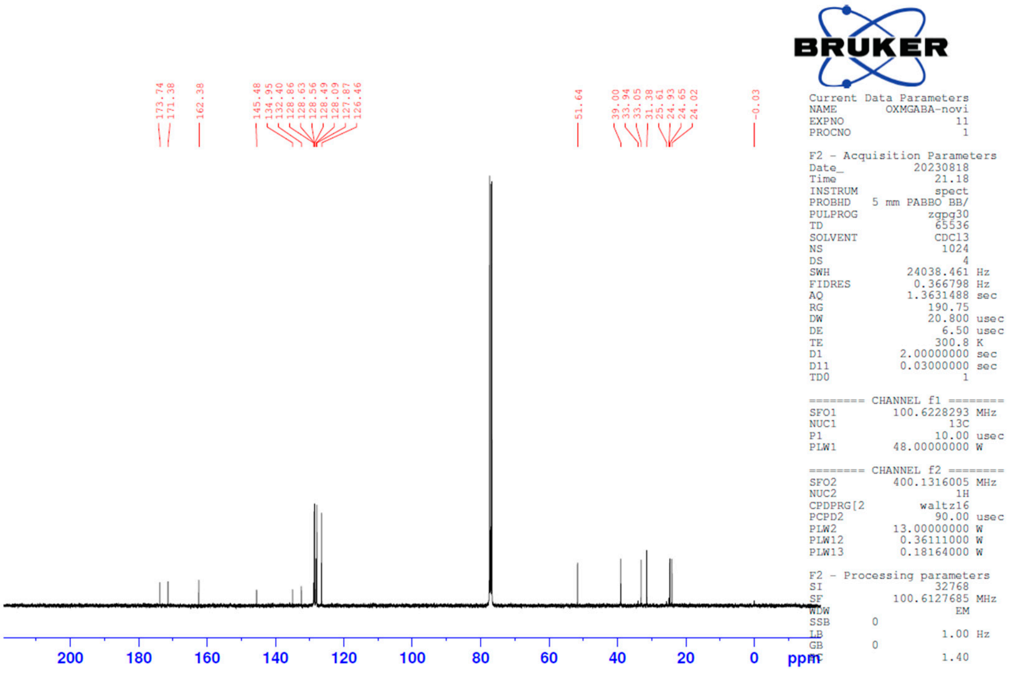


**Figure S9**. $^{1}H$ NMR spectrum (a) and $^{13}C$ NMR spectrum (b) of compound **3**.

a)

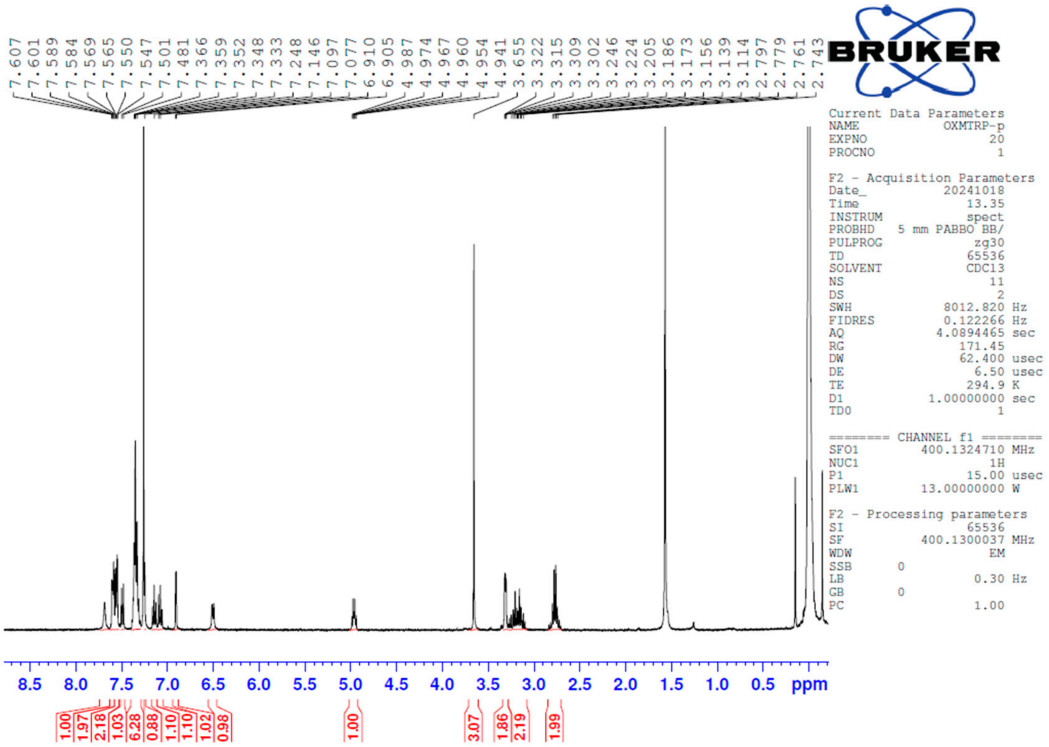


b)

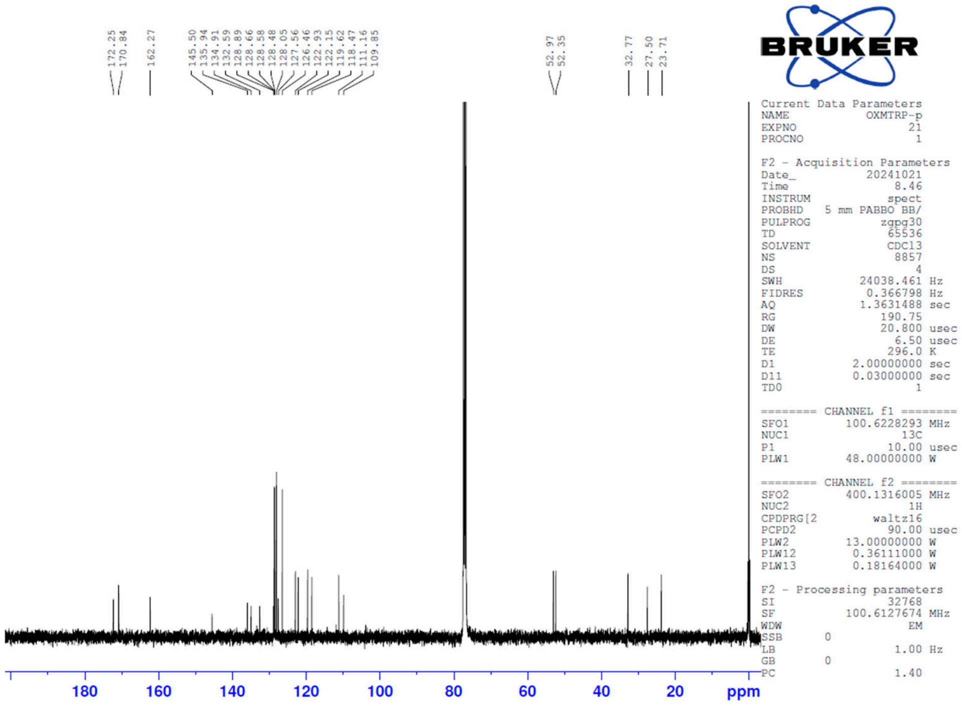


**Figure S10**. $^{1}H$ NMR spectrum (a) and $^{13}C$ NMR spectrum (b) of compound **4**.

a)

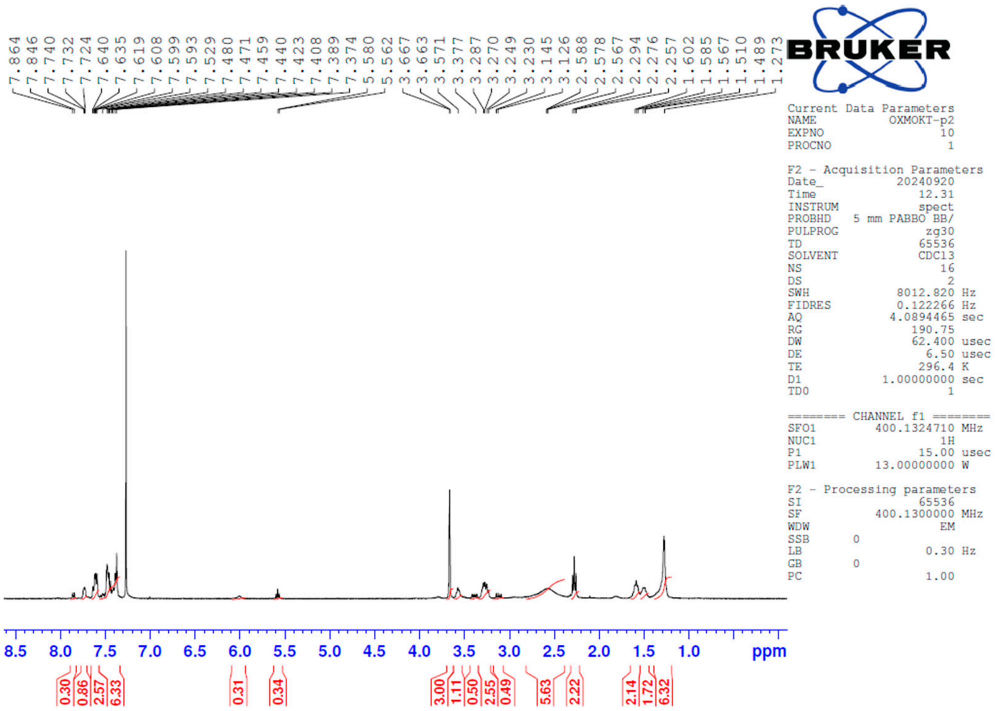

b)

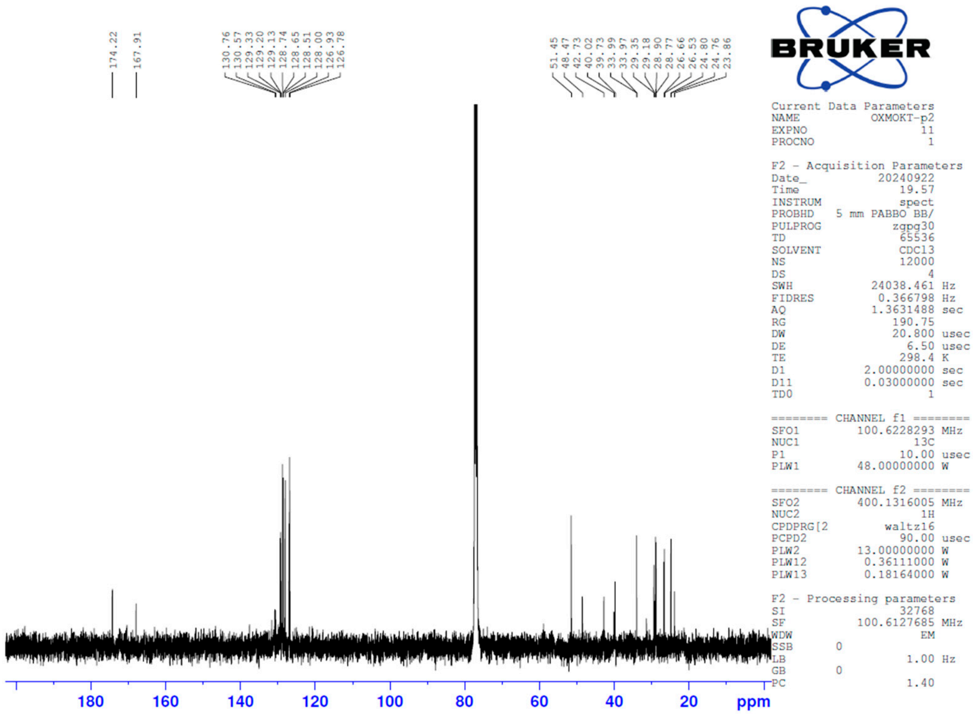

**Figure S11**. $^{1}$H NMR spectrum (a) and $^{13}$C NMR spectrum (b) of compound **5**.

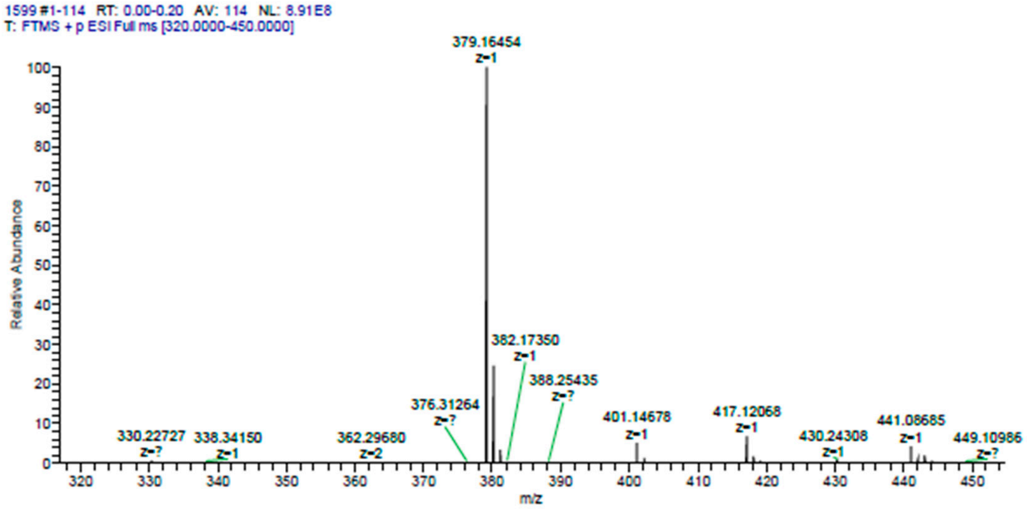


Zoomed spectra

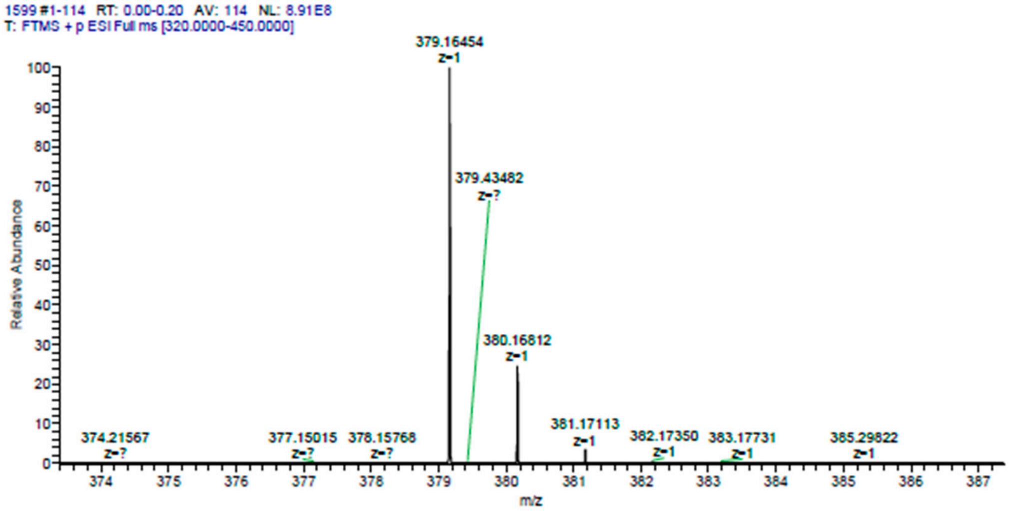


| Exact mass | Observed mass | Observed ion type | Error (ppm) |
|---|---|---|---|
| 379.16523 | 379.16454 | $[M+H]^+$ | 1.82 |

**Figure S12**. HRMS spectrum of compound **1**.

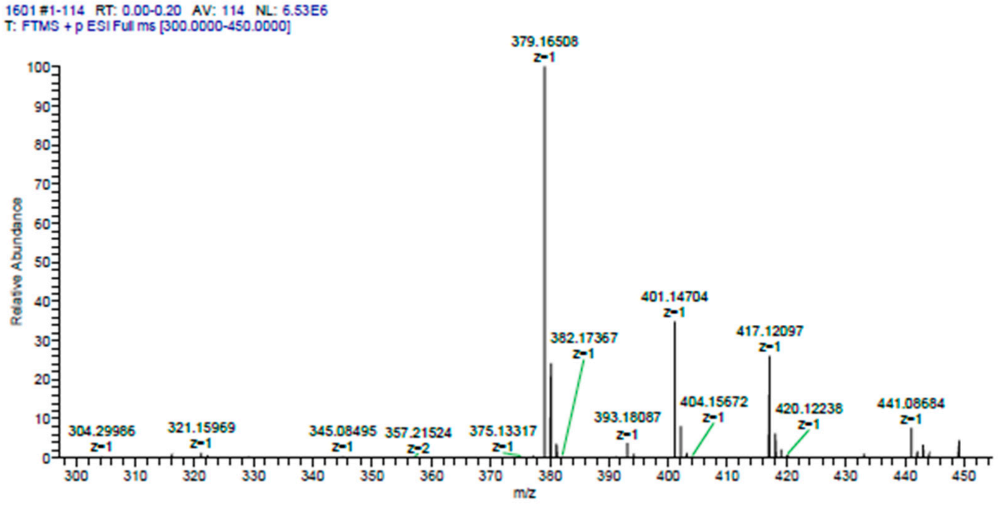


## Zoomed spectra

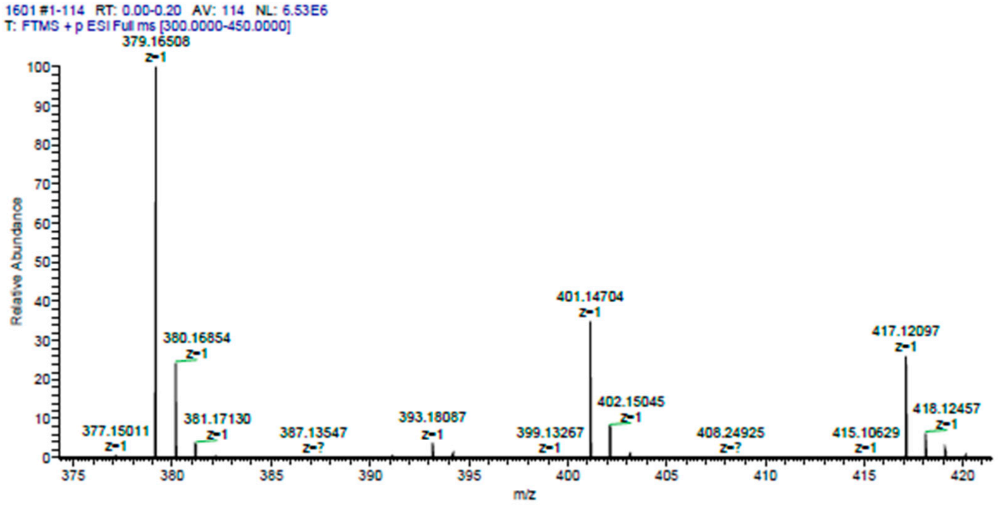


| Exact mass | Observed mass | Observed ion type | Error (ppm) |
|---|---|---|---|
| 379.16523 | 379.16508 | $[M+H]^+$ | 0.40 |

**Figure S13**. HRMS spectrum of compound **2**.

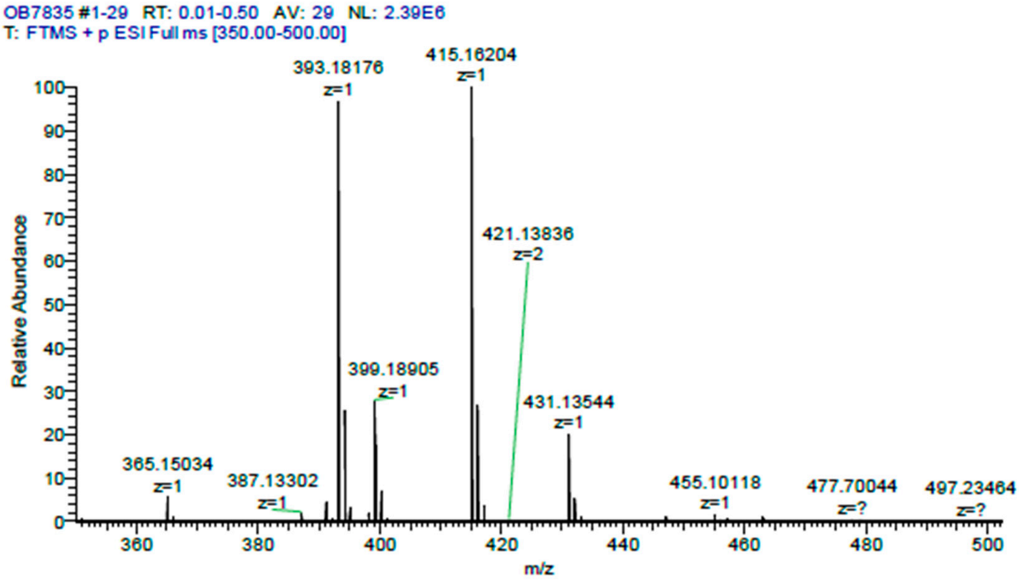


Zoomed spectra

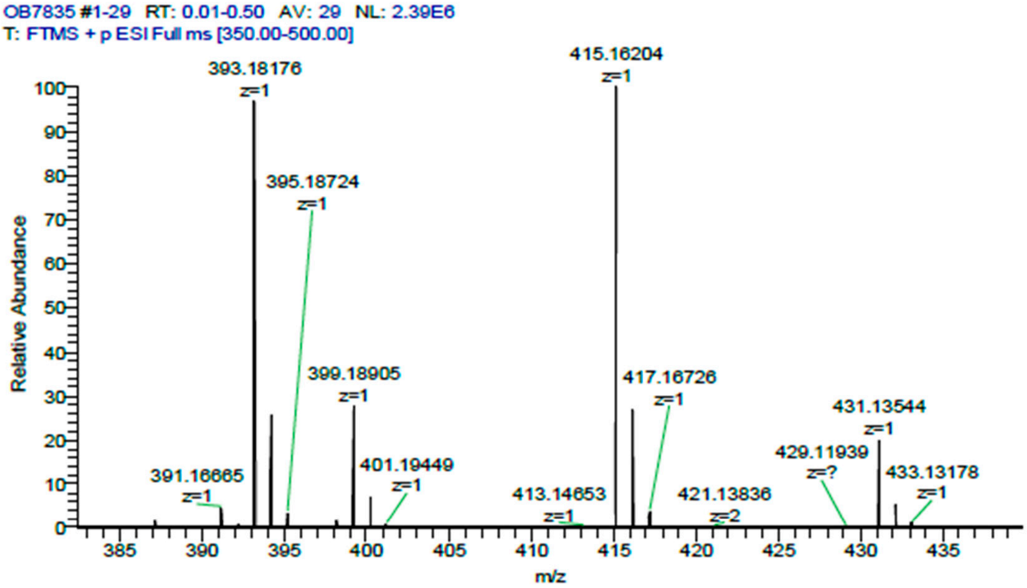


| Exact mass | Observed mass | Observed ion type | Error (ppm) |
|---|---|---|---|
| 393.18088 | 393.18176 | $[M+H]^+$ | 2.24 |
| 415.16283 | 415.16204 | $[M+Na]^+$ | 1.90 |

**Figure S14**. HRMS spectrum of compound **3**.

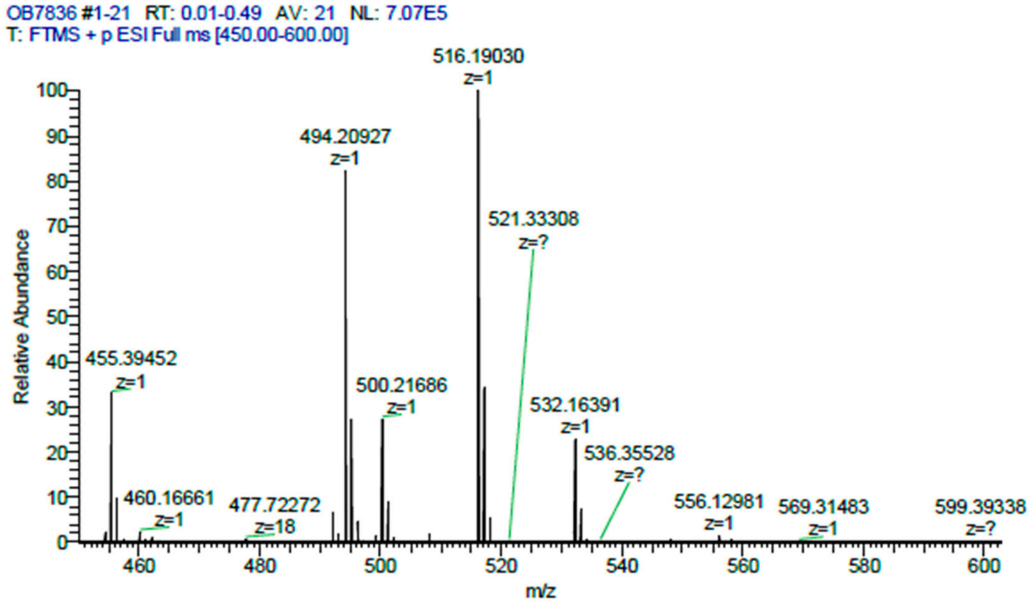


Zoomed spectra

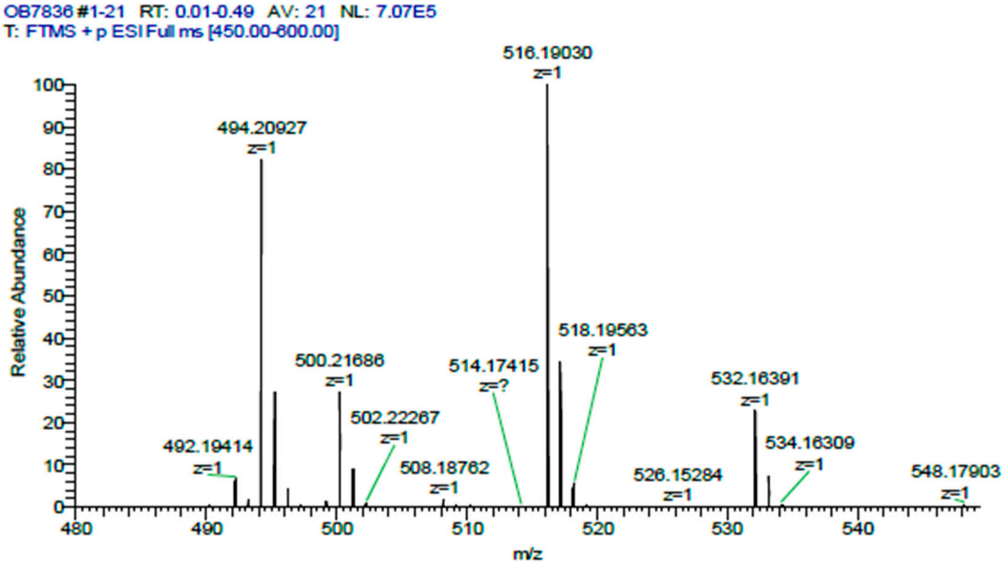


| Exact mass | Observed mass | Observed ion type | Error (ppm) |
|---|---|---|---|
| 494.20743 | 494.20927 | $[M+H]^+$ | 3.72 |
| 516.18938 | 516.19030 | $[M+Na]^+$ | 1.78 |

**Figure S15**. HRMS spectrum of compound **4**.

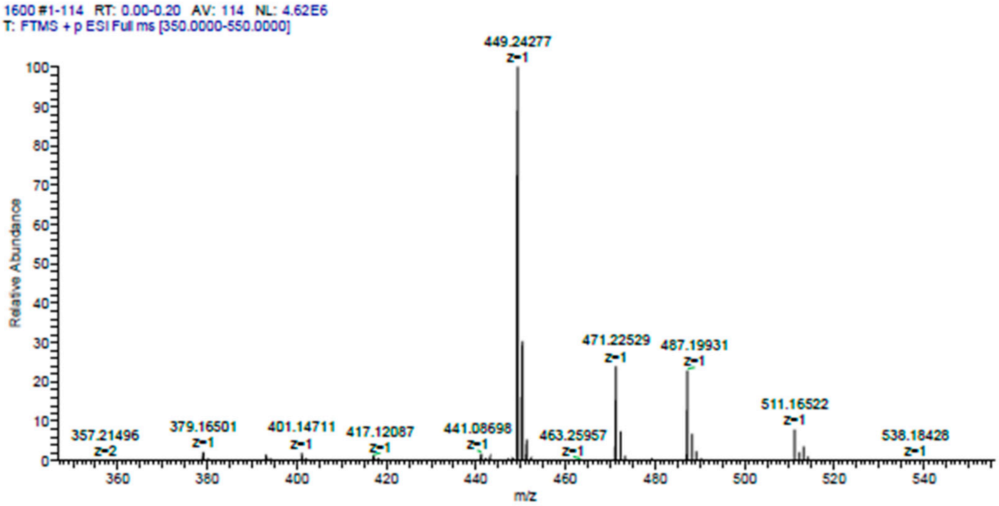


Zoomed spectra

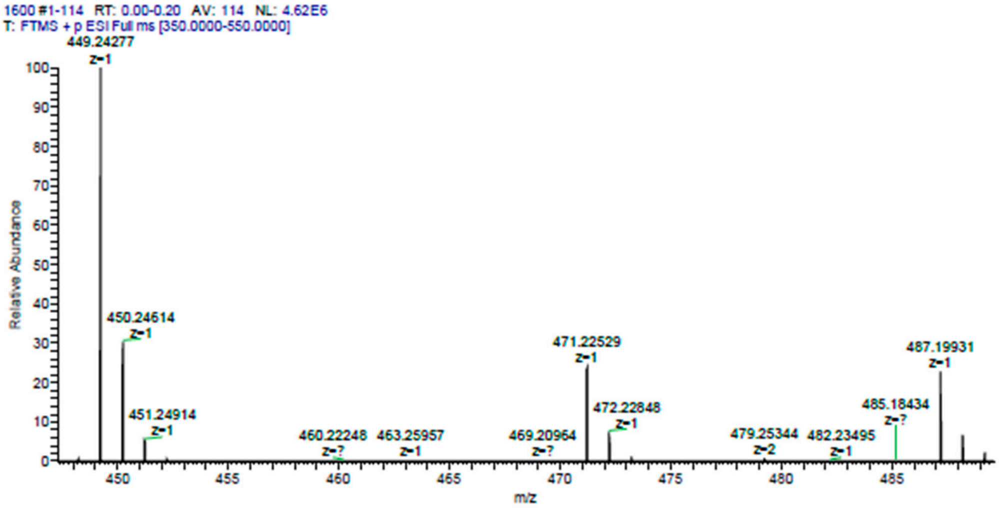


| Exact mass | Observed mass | Observed ion type | Error (ppm) |
|---|---|---|---|
| 449.24348 | 449.24277 | $[M+H]^+$ | 1.58 |

**Figure S16**. HRMS spectrum of compound **5**.
